# Hypoxia coordinates the spatial landscape of myeloid cells within glioblastoma to affect survival

**DOI:** 10.1126/sciadv.adj3301

**Published:** 2024-05-17

**Authors:** Michael J. Haley, Leoma Bere, James Minshull, Sokratia Georgaka, Natalia Garcia-Martin, Gareth Howell, David J. Coope, Federico Roncaroli, Andrew King, David C. Wedge, Stuart M. Allan, Omar N. Pathmanaban, David Brough, Kevin N. Couper

**Affiliations:** ^1^ Geoffrey Jefferson Brain Research Centre, Manchester Academic Health Science Centre, Northern Care Alliance NHS Foundation Trust, University of Manchester, Manchester, UK.; ^2^ Lydia Becker Institute of Inflammation and Immunology, University of Manchester, Manchester, UK.; ^3^ Division of Neuroscience, University of Manchester, Manchester, UK.; ^4^ Division of Informatics, Imaging and Data Sciences, University of Manchester, Manchester, UK.; ^5^ Department of Statistics, University of Oxford, Oxford, UK.; ^6^ Flow Cytometry Core Research Facility, University of Manchester, Manchester, UK.; ^7^ Manchester Centre for Clinical Neurosciences, Manchester, UK.; ^8^ Division of Cardiovascular Sciences, University of Manchester, Manchester, UK.; ^9^ Manchester Cancer Research Centre, University of Manchester, Manchester, UK.

## Abstract

Myeloid cells are highly prevalent in glioblastoma (GBM), existing in a spectrum of phenotypic and activation states. We now have limited knowledge of the tumor microenvironment (TME) determinants that influence the localization and the functions of the diverse myeloid cell populations in GBM. Here, we have utilized orthogonal imaging mass cytometry with single-cell and spatial transcriptomic approaches to identify and map the various myeloid populations in the human GBM tumor microenvironment (TME). Our results show that different myeloid populations have distinct and reproducible compartmentalization patterns in the GBM TME that is driven by tissue hypoxia, regional chemokine signaling, and varied homotypic and heterotypic cellular interactions. We subsequently identified specific tumor subregions in GBM, based on composition of identified myeloid cell populations, that were linked to patient survival. Our results provide insight into the spatial organization of myeloid cell subpopulations in GBM, and how this is predictive of clinical outcome.

## Teaser

Multimodal mapping reveals that the spatial organization of myeloid cells in glioblastoma affects the duration of survival.

## INTRODUCTION

Glioblastoma (GBM), the most common and aggressive type of brain tumor, is almost universally fatal, with a median average survival time of 12 to 18 months. Despite the tremendous advances in treatment of other cancers, the standard-of-care treatment for GBM—surgical resection followed by chemoradiotherapy—has remained unchanged for decades (*1*). To date, GBM has proven largely refractive to immunotherapies highly effective in other cancers (*2*). Consequently, there is a critical need to better understand the unique immunobiology of GBMs to develop new effective treatments for this devastating disease.

Myeloid cells (which include monocytes, macrophages, and tissue resident microglial cells) are major components of GBM, constituting 30 to 40% of all cells found within the GBM tumor microenvironment (TME) (*3*, *4*). Myeloid cells are believed to play major roles in promoting GBM progression and treatment resistance, including impairing response to radiotherapy and immunotherapy. As such, they are viewed as attractive targets for novel treatments for GBM, with preclinical data supporting the benefit of macrophage modulation (*3*). However, the myeloid cell compartment is extremely heterogeneous in GBM, being able to adopt a spectrum of proinflammatory and suppressive states (*4*, *5*) with immunosuppressive phenotypes being associated with worse outcome (*6*–*11*).

At present, we have limited knowledge of where the different myeloid cell populations compartmentalize in the complex TME of GBMs, and what tissue factors may shape their positioning and differentiation. This is partly due to the limitations of bulk-based or cell suspension–based methods for studying the GBM TME, which cannot simultaneously capture the spatial and phenotypic heterogeneities seen in myeloid cells in GBM (*4*). Spatial heterogeneity arises in GBM due to variation in the degree of tumor cell infiltration into healthy tissue and due to emergence of hallmark features of GBM such as hypoxia-induced necrosis and microvascular proliferation that differentiate it from lower-grade tumors (*12*). Further spatial heterogeneity emerges from the proliferation of specific tumor subclones within different parts of the same tumor (*12*, *13*) and macroscopically in terms of blood flow and tissue perfusion (*14*). Therefore, there are established spatial features within GBM that may imprint myeloid cell heterogeneity and cell compartmentalization.

Here, we have combined high-dimensional imaging mass cytometry (IMC) and deconvolution of spatial transcriptomic (ST) datasets to map the myeloid cell compartment in the TME of GBM. Orthogonal validation identified distinct populations of myeloid cells in GBM, with subsets of macrophages and microglia that differed in abundance between areas from the edge and core of the tumor. We found clear compartmentalization of myeloid populations, with cells of a similar phenotype clustering together (with macrophages showing particularly strong clustering behavior) and segregating cells of dissimilar phenotypes. The spatial landscape of myeloid cells appeared to be modulated by several putative factors, including cellular interactions (with myeloid, tumor, and vascular cells), regional chemokine signaling, and, predominantly, tissue hypoxia. Hypoxic niches were preferentially occupied by immunosuppressive macrophages and infiltrating monocytes. Crucially, we identified an association between the transcriptomic signature of specific myeloid cell environments and GBM survival, indicating that the spatial arrangement of specific myeloid cell populations is an important determinant of GBM outcome.

## RESULTS

### IMC captures the diverse myeloid states present in GBM

**Fig. 1.**
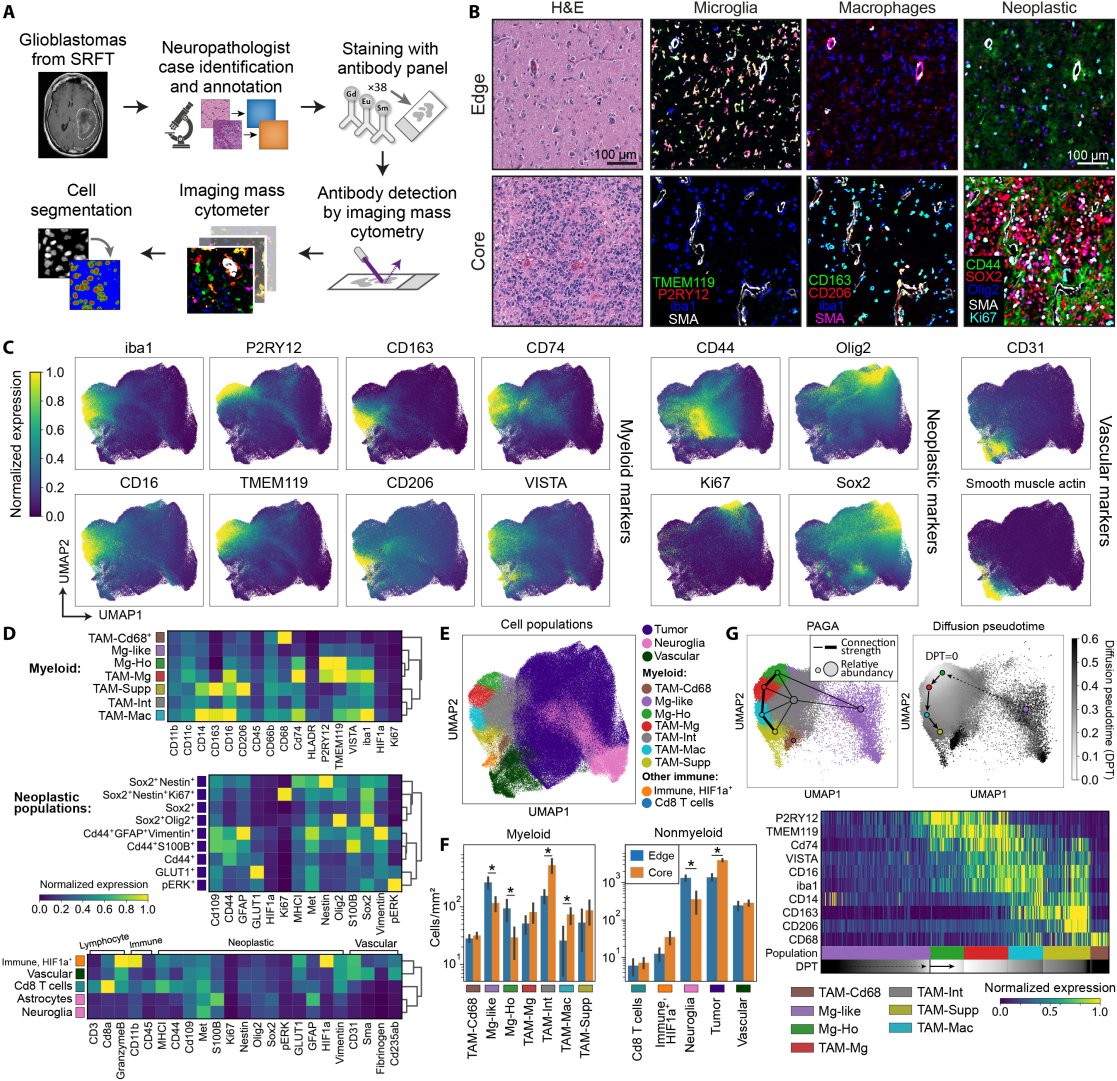
Characterization of cell populations present in the GBM TME using IMC. (**A**) Overview of the GBM patient samples obtained from Salford Royal Foundation Trust (STFT) and IMC workflow. (**B**) Representative IMC and H&E images from GBM cases taken from either the edge or core of the tumors, visualizing key microglial, macrophage, and neoplastic markers. (**C**) UMAPs visualizing the single-cell data acquired from the IMC workflow, demonstrating the distribution of the myeloid, neoplastic, and vascular markers over all cells. Each marker is normalized to the 99th percentile of its expression. (**D**) Heatmaps showing the mean marker expression for the populations identified in the single-cell IMC data using Leiden clustering. (**E**) UMAP showing the labeled cell populations resulting from Leiden clustering. (**F**) Comparison of the abundances of the different myeloid and nonmyeloid populations between the edge and core regions of the tumors. *P < 0.05, groups compared by multiple linear regression. Data shown as mean ± SEM. (**G**) Diffusion pseudotime and PAGA analysis demonstrating a pathway of myeloid differentiation starting at homeostatic microglia (Mg-Ho), through to proinflammatory activation microglia (TAM-Mg), proinflammatory macrophages (TAM-Mac), and ultimately to immunosuppressive myeloid cells (TAM-Supp).

Primary IDH (Isocitrate dehydrogenase 1)–wild-type (WT) GBMs were selected from the Department of Cellular Pathology at Salford Royal Hospital (Table 1), and edge and core regions of the tumors were annotated by a neuropathologist via histological assessment of hematoxylin and eosin (H&E)–stained sections. Tissue microarrays (TMAs) were subsequently generated (24 mm by 2.25 mm), and sections were stained with a panel of 37 metal-conjugated antibodies, followed by data acquisition on a Hyperion IMC. Nuclear-based cell segmentation was used to extract the single-cell expression of each marker in the staining panel for downstream analysis (Fig. 1A), with accuracy of segmentation confirmed against manual annotation via Jaccard analysis (fig. S1A) (*15*).

**Table 1.** Clinical characteristics of samples for IMC.

Case	Site	Age at surgery	Sex	*IDH* status	*ATRX *status	*MGMT *meth ylation status	Edge regions	Core regions
1	Left temporal	63	F	WT	WT	Not performed	2	1
2	Left temporal	73	F	WT	WT	Hypermethyl ated	1	2
3	Right parietal	63	F	WT	WT	Unmethylated	2	1
4	Left temporal	53	M	WT	WT	Unmethylated	2	1
5	Left parietal	54	F	WT	WT	Hypermethyl ated	2	1
6	Left frontal	62	F	WT	WT	Hypermethyl ated	2	1
7	Left temporal	73	M	WT	WT	Unmethylated	1	2
8	Left cingulate	62	M	WT	WT	Not performed	3	0

IMC allowed us to simultaneously identify and contrast the components and spatial organization of the TME within the edge and core regions in situ. Given the role of myeloid cells in GBM (*5*), we designed our antibody panel and subsequent analyses to capture the diversity of myeloid cell populations found within GBM. IMC allowed us to putatively identify microglia (iba1^+^, TMEM119^+^, and P2RY12^+^) and tumor-associated macrophages (iba1^+^ and CD163^+^) based on their morphology and marker expression, as well as cells positive for markers of central nervous system (CNS) progenitor cells and cellular proliferation often associated with neoplastic cells (CD44, SOX2, Olig2, and Ki67) (Fig. 1B). Representative images suggested the abundance of microglia, and TAMs varied between the edge and core of the tumor, as previously reported (*9*, *16*–*18*).

To understand the expression patterns of the analyzed molecules within the single cells segmented within our dataset, we first visualized their distribution using Uniform Manifold Approximation and Projection (UMAP). This showed clear segregation between cells expressing myeloid, neoplastic, and vascular makers (Fig. 1C and fig. S1B). Within the region corresponding to myeloid cells, there was separation between cells with highest expression for markers of microglia (TMEM119^+^ and P2RY12^+^) and tumor-associated macrophages (iba1^+^ and CD163^+^). However, the lack of a discrete boundary between microglia and TAM cell clusters, and the distribution pattern of other myeloid markers (CD74, CD16, VISTA, and CD206), suggested the presence of multiple closely and distantly related myeloid subpopulations in the GBM tumor. Similarly, neoplastic markers were heterogeneously expressed throughout the neoplastic compartment of the UMAP, likely corresponding to distinct neoplastic populations.

To identify the cell populations present in our IMC data, Leiden clustering was performed (*19*), resulting in profiling of 21 distinct myeloid and nonmyeloid populations (Fig. 1, D and E, and fig. S1C). Within these, seven were identified as myeloid populations (fig. S1C), nine populations were identified as tumor cells, two corresponded to neuroglia (astrocytes and other CNS cells), three to nonmyeloid immune populations (lymphocytes and a HIF1a^+^ immune population), and one to vascular cells (Fig. 1E). All populations were confirmed by visualizing them in the tissue (fig. S1D). The myeloid populations were detectable across cases, although varied in their proportions (fig. S1E). Two populations were identifiable as microglia. The first showed higher expression of the homeostatic microglial marker P2RY12 (Mg-Ho; homeostatic microglia) and the second having a more proinflammatory phenotype (TAM-Mg; tumor-associated microglia), with lower P2RY12 and increased expression of markers of proinflammatory activation (iba1^high^, VISTA^+^, CD16^+^, and CD74^high^). Myeloid cells high for CD74 have previously been associated with a proinflammatory (M1) phenotype in GBM (*20*), and high expression of VISTA has been reported in activated macrophages (*21*). Two populations were identified as tumor-associated macrophages (CD163^+^), with one having a more proinflammatory phenotype (TAM-Mac; tumor-associated macrophages) and moderate expression of TMEM119. The other TAM population had comparatively lower expression of CD14 and CD16 but high expression of CD206 (TAM-Supp; immunosuppressive myeloid), a phenotype which has suggested to reflect immunosuppressive myeloid cells in GBM (*11*, *18*, *22*, *23*). Besides populations with defined phenotypes, we also found a sizeable population of intermediate TAM/microglial population without a canonical microglia or macrophage signature, as has previously been reported (*24*, *25*). This population was confirmed as not being a consequence of poor segmentation (fig. S1, A and D). We also found a population of myeloid cells with low expression of myeloid markers relative to other myeloid cells, which may represent cells with low activation (Mg-like; microglia-like), and a population which were only positive for CD68 (TAM-Cd68). Notably, we could not differentiate monocytes or dendritic cells as distinct populations utilizing this panel, although recent reports suggest that dendritic cells make up <1% of total myeloid cells in the GBM TME (*22*, *26*). Although clustering did identify putatively different tumor cell populations, our panel did not robustly distinguish the neoplastic subtypes known to be present in GBM (*27*). As such, to facilitate downstream analyses, we merged tumor populations into a single population. Cells classified as neuroglia did not express markers that would otherwise identify them as myeloid, tumor, or vascular cells and thus likely represent a mixed population of glia and other CNS-resident cells. On the basis of their high expression of granzyme B and CD11b, we hypothesize that HIF1a^+^ immune population could be neutrophils.

### The abundance of myeloid populations varies in the different GBM regions

Previous studies have reported that microglia and macrophages are preferentially found at the periphery of the tumor or the core of the tumor, respectively (*9*, *16*–*18*). We therefore calculated the abundance of the seven myeloid subpopulations and nonmyeloid cells, within the histologically validated edge and core regions of the tumor (Fig. 1F). We found a significant increase in the numbers of homeostatic microglia and microglial-like cells in the edge and an increase in intermediate and proinflammatory populations (TAM-Int and TAM-Mac) in the core. Hierarchical clustering of regions, based on relative myeloid abundances, further indicated that edge and core regions contained distinct myeloid cell compartments (fig. S2A). We also saw an increase in neuroglial cells in the tumor edge and, as expected, an increase in tumor cells in the core. Correlating the abundances of the various populations within each region of interest showed distinct clusters of positively and negatively correlated populations (fig. S2B). For example, microglia (Mg-Ho and Mg-like) and neuroglial cell abundances correlated positively with each other but negatively correlated with TAM populations (TAM-Mac and Tam-Supp) and tumor cell numbers. Notably, although there were differences in abundances of myeloid subpopulations between core and edge regions, most populations were found in all regions analyzed (Fig. 1F and fig. S2C). Together, these analyses suggest that although there may be broader features of the TME that promote the accumulation of microglia versus tumor-associated macrophages, there may be more specific local features present in regions which drive the accumulation of specific myeloid subpopulations.

### Myeloid populations exist on a spectrum of differentiation and activation states in GBM

Our IMC data, along with published single-cell sequencing studies, demonstrate that myeloid cells exist in a spectrum of activation states in GBM (*7*, *18*) with the polarization of myeloid cells toward either microglia or TAMs being the most prominent (*22*, *28*, *29*). However, the exact ontogeny of the identified myeloid populations in GBM remains unclear (*4*). We therefore used partition-based graph abstraction (PAGA) analysis to understand the relationships between the seven myeloid populations identified by IMC (Fig. 1G). This showed strong connectivity and therefore a pathway of differentiation between four myeloid populations: homeostatic microglia (Mg-Ho), proinflammatory microglia (TAM-Mg), proinflammatory macrophages (TAM-Mac), and immunosuppressive myeloid cells (TAM-Supp). As microglia (Mg-Ho) are the brain resident immune cells, we then assessed the differentiation from this native state using diffusion pseudotime analysis. Plotting pseudotime against the route of differentiation defined by PAGA demonstrated that microglial markers are lost in favor of activation markers (VISTA, CD74, CD16, and iba1 up-regulation) and eventually transitioning into an immunosuppressive phenotype characterized by up-regulation of CD163 and CD206 (*18*, *23*). Together, these analyses suggest that endogenous microglia may differentiate into tumor-associated macrophages and that the primary pathway through which this occurs is via a state of proinflammatory activation. An alternate pathway by which cells first transition through intermediary state (TAM-Int) before differentiating into immunosuppressive TAMs is less well supported by the PAGA and pseudotime analyses (fig. S2D). However, as we could not identify monocytes as a defined population, these pathways only address how endogenous microglia differentiate into tumor-associated macrophages. There is an established route through which infiltrating monocytes differentiate into immunosuppressive (CD163^+^ CD206^+^) tumor-associated macrophages (*17*, *18*, *22*) that likely operates in parallel that we cannot address in our IMC data.

### Microglia and macrophages show conserved patterns of compartmentalization in GBM

**Fig. 2.**
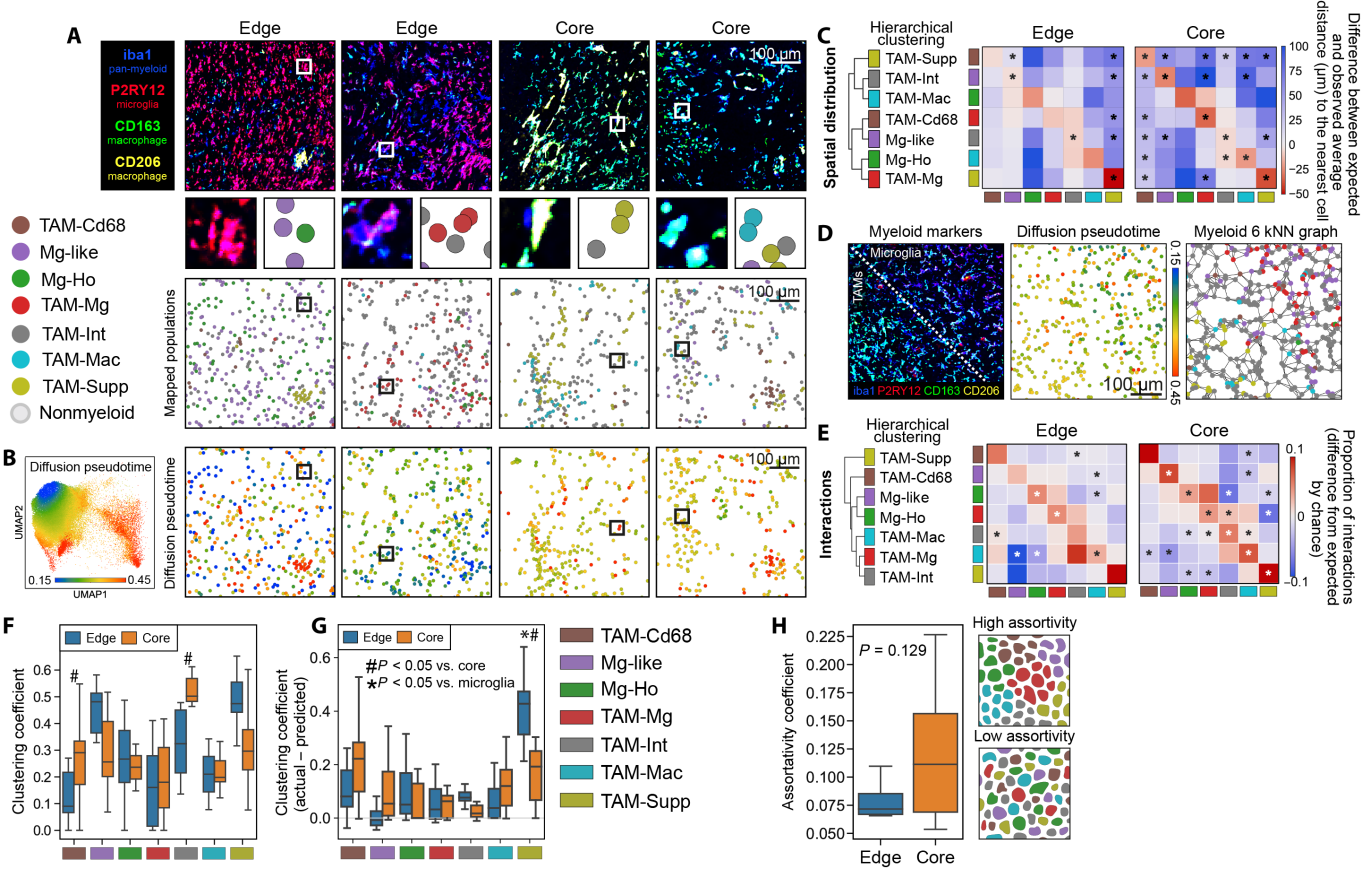
Myeloid cells exhibit high homotypic and low heterotypic clustering behavior in GBM. (**A**) Mapping of the myeloid populations identified from single-cell analyses to their locations in the TME in edge and core regions. (**B**) Diffusion pseudotime showing how differentiation away from a homeostatic microglial phenotype relates to myeloid cell positioning. (**C**) Spatial distribution analysis which shows whether populations are closer or further away from each other in the TME than would be expect by chance. (**D**) IMC image showing separation of microglia (P2RY12^+^) and macrophages (CD163^+^ and CD206^+^), alongside the position of the cells on the microglia-macrophage diffusion pseudotime axis, and how the cells are connected in the six–nearest neighbors (kNN) analyses. (**E**) Proportion of cellular interactions made by myeloid populations when each myeloid cell is connected to its six nearest neighbors. Hierarchical clustering of the interactions shows that cells with a similar phenotype also have similar proportions of interactions with other populations. (**F**) Clustering coefficient of the different populations in the edge and core of the tumor. (**G**) Correction of clustering coefficients for differences in cell abundance, in which a positive value suggests that cells are clustering at a greater rate than expected by chance. (**H**) Comparison of assortativity of myeloid populations in edge and core regions. Comparison made by Wilcoxon test with Benjamini-Hochberg correction (C and E), multiple linear regressions, corrected for multiple comparisons by Holm-Šídák (F and G), or Mann-Whitney *U* test (H). Box plots show range, interquartile range, and median of data (F to H).

Although we observed different abundances of myeloid populations between core and edge regions, our data clearly identified that multiple different myeloid cell populations were present within the same areas of the tumor. This raised the question whether myeloid cell compartmentalization in GBM is stochastic, or whether there are deterministic cellular and environmental drivers of myeloid positioning and function. To investigate this, the previously identified myeloid populations (Fig. 1D) were mapped back to their locations in the tumor (Fig. 2A). This revealed that different myeloid populations had different distributions in the TME, with some populations appearing to cluster in specific areas (e.g., TAM-Supp), others aggregated more loosely (e.g., TAM-Int), whereas others appeared more evenly distributed in the TME (e.g., Mg-Ho). Qualitatively, these patterns seemed conserved between the core and edge of the tumor. Notably, myeloid cells appeared to spatially associate with other myeloid cells in a similar position on the microglial-TAM differentiation axis defined by diffusion pseudotime (Fig. 2B, pseudotime defined in Fig. 1G).

To prove whether there was a preference for myeloid populations to localize close to or away from other populations in the TME, we quantified whether the observed distance between myeloid populations was significantly different from the distance predicted by a random distribution of cells (Fig. 2C). Hierarchical clustering of the resulting data demonstrated that microglial (Mg-like, Mg-Ho, and TAM-Mg) and macrophage subpopulations (TAM-Mac and TAM-Supp) have distinct patterns of spatial distribution. Specifically, microglial and macrophage populations appeared to show a preference for spatial segregation, with macrophage populations associating with one another and localizing away from microglia and microglia enriching with other microglia and localizing away from macrophages. All populations had the greatest spatial enrichment with themselves. In some populations (TAM-Cd68, TAM-Int, and Mg-like), the difference between the expected and observed distances to other populations was closer to zero, although often still significant. This suggests that these populations are closer to a stochastic distribution, being more randomly and evenly distributed throughout the TME. As the overall pattern of interactions was broadly similar between edge and core regions, it suggests a conservation of the propensity of myeloid cells with a similar phenotype to spatially associate together even in the context of different cell abundances and environments throughout the tumor. To assess whether the spatially associated myeloid populations were interacting in the GBM TME, myeloid cells in each region were subsequently expressed as networks, where each cell was connected to its six nearest neighbors (Fig. 2D and fig. S3A). This analysis supported our previous observations (Fig. 2C), showing that myeloid populations most significantly interact with themselves, interact to some degree with phenotypically similar populations, and tend not to interact with dissimilar populations (Fig. 2E). As with previous analyses, this pattern of behavior was similar between edge and core regions, suggesting that this behavior is conserved throughout the tumor.

The tendency of myeloid cells to spatially associate with cells of the same type was investigated by measuring each populations’ clustering coefficient (Fig. 2F). Clustering coefficient is a descriptive statistic of network properties in which a high value designates that a population forms densely interconnected clusters (*30*), and a low value suggests cells of that population are weakly connected and more loosely clustered in the TME. In this initial analysis, all myeloid populations showed a similar propensity for clustering, with significantly higher clustering in TAM-Cd68 and TAM-Int populations in the core. These clustering values suggest that most myeloid populations form small, weakly connected clusters in the TME. As more abundant populations could be clustering purely by chance, we then corrected clustering coefficients for differences in population abundance between regions. This showed that almost all populations showed more clustering than would be expected by chance (Fig. 2G). Notably, TAM-Supp cells showed significantly denser clustering in the edge. The pattern of clustering was otherwise conserved between edge and core regions. Measuring the assortativity [a descriptive statistic of the tendency of populations in a network to connect to populations of the same type over different populations (*31*)] similarly suggested that cells showed a weak but positive preference to connect to cells of the same population and that this was similar between edge and core regions (Fig. 2H). Overall, these data demonstrate that different myeloid populations segregate and form loose homotypic clusters in the TME and that the biological drivers of this segregation are mostly independent of broader location in the edge or core of the tumor.

### The positioning of myeloid cell populations is affected by tumor, neuroglial, and vascular interactions in the GBM TME

**Fig. 3.**
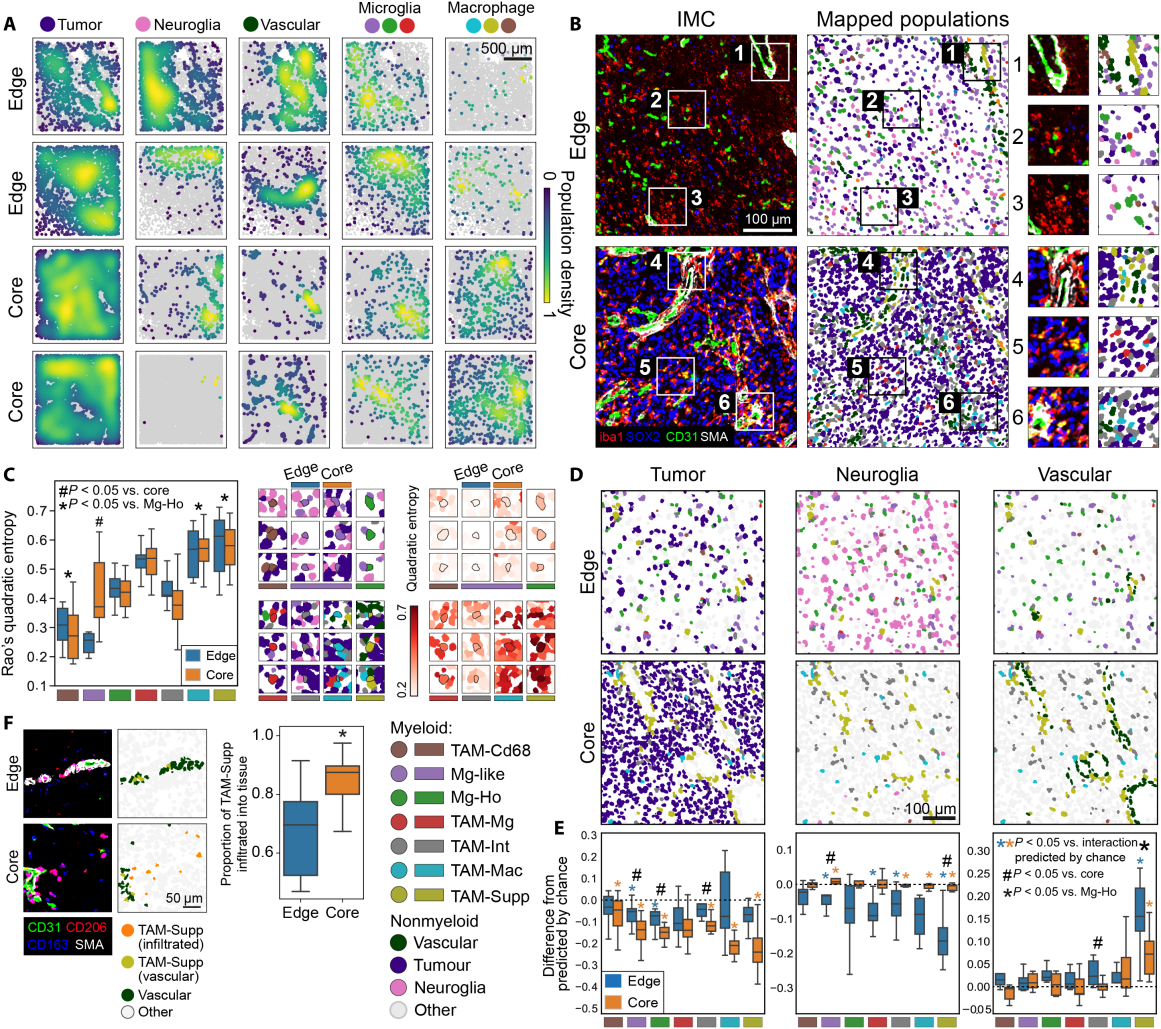
Myeloid cell interactions with tumor, neuroglial, and vascular cells in GBM. (**A**) Spatial density of different cell populations (calculated by Gaussian kernel density estimation) in the TME. (**B**) Myeloid cells mapped alongside tumor, neuroglial, and vascular cells in representative edge and core regions. (**C**) Quadratic entropy for the myeloid populations in the edge and core of the tumor. This quantifies how heterogeneous a cell is with respect to its interacting cell, with high values indicating that a cell interacts with several cells with different phenotypes. (**D**) Representative examples of myeloid populations mapped alongside nonmyeloid populations. (**E**) Difference between the observed rate of cell-to-cell interaction data (see fig. S3C) and the rate of interactions predicted by chance. This analysis corrects for the established differences in abundances of the myeloid and nonmyeloid cells between different regions. (**F**) Comparison between edge and core regions in the rate of infiltration of TAM-Supp cells into the parenchyma, defined here as being 10 μm away from nearest vascular cell. Comparisons made by linear mixed models (B, C, and E) or Wilcoxon tests (E) with Holm-Šídák corrections or Mann-Whitney *U* test (F). Box plots show range, interquartile range, and median of data (C, E, and F).

Our data suggest that myeloid cells are not randomly distributed in the GBM TME. We hypothesized that this myeloid compartmentalization could be driven by myeloid cells interacting with other highly abundant nonmyeloid cells in the TME, such as tumor (SOX2), vascular [CD31 and smooth muscle actin (SMA)], and neuroglial cells (defined in Fig. 1). Mapping the relative densities of these different populations demonstrated macroscopic regionalization of tumor and neuroglial cells within the core and edge tumor regions and of myeloid populations when broadly divided into microglial and macrophage populations (Fig. 3A). Mapping the populations at the cellular level qualitatively suggested potential associations between specific myeloid subpopulations and neuroglial, vascular, and tumor cells (Fig. 3B).

To quantify the different environments in which the different myeloid cell populations were found, we used a local measure of Rao’s quadratic entropy (Fig. 3C). This measures the phenotypic diversity between each myeloid cell and the cells (nonmyeloid and myeloid) with which it interacts, with highly scoring cells interacting with several cells with heterogeneous phenotypes. This demonstrated that macrophage populations (TAM-Mac and TAM-Supp) had significantly greater quadratic entropy than microglia. While we have previously established that myeloid populations cluster in the tissue (Fig. 2), these analyses further suggest that macrophages form clusters in more cellularly heterogeneous areas of the tumor, interacting with multiple nonmyeloid cells with diverse phenotypes. In comparison, microglia have less diversity in their interactions with other nonmyeloid cells. The only difference between edge and core was in Mg-like cells, where cells in the core had significantly greater quadratic entropy. This suggests that the local cellular organization of nonmyeloid cells influences myeloid cell localization in a way that is conserved throughout the tumor, so that macrophages preferentially cluster in cellularly dense and phenotypically diverse areas of the tumor.

Quantification of interactions between myeloid cells and tumor, neuroglial, and vascular cells demonstrated that almost all myeloid populations had significantly greater proportion of their cellular interactions with tumor cells in the core and significantly greater interactions with neuroglial cells in the edge (Fig. 3D and fig. S3C). While this suggested that there were distinct neoplastic and neuroglial influence on myeloid cell positioning in edge and core, these effects could be affected by established differences in myeloid, tumor, and neuroglial cell abundances in different regions and cases (Fig. 1F). For example, highly abundant populations may be colocalizing and so appear to be interacting, purely by chance. Once we corrected rates of cellular interactions between myeloid cell populations with nonmyeloid cells for differences in cellular abundances in the edge and core regions, myeloid populations in both the edge and core showed significantly less interactions with tumor and neuroglial cells than would be expected by chance (Fig. 3E). This could suggest that myeloid cells are actively avoiding interacting with tumor cells or are preferentially responding to other cues, which makes myeloid-tumor interactions less common than would be expected by chance.

TAM-Mac and TAM-Supp cells showed the greatest avoidance (i.e., a propensity to have less interactions than would be expected) with tumor cells, particularly in the core. Furthermore, abundance-corrected interactions suggested that myeloid populations also avoided interacting with neuroglial cells, particularly in the edge, although also to a lesser extent in the core. The greatest avoidance of neuroglial cells was by TAM-Supp cells in the edge. In corrected values, TAM-Supp cells continued to be the only population with a significant interaction with vascular cells in both edge and core cases, but differences in the strength of association were identified in the two different tumor regions (Fig. 3E). These findings were corroborated using cross–pair correlation function (PCF) analysis, a statistical approach to assess cell-to-cell interactions that considers differences in population abundances (*32*, *33*). This similarly found an association of TAM-Supp cells with the vasculature and less interactions than predicted by chance between myeloid and tumor cells, which was consistent between the edge and core of the tumor (fig. S3D). Similar analyses were performed on CD8^+^ T lymphocytes and the immune HIF1a^+^ populations, finding that HIF1^+^ cells (that we hypothesize could be neutrophils) were vascular associated (fig. S3E). CD8^+^ T cells did not show enriched interactions with any myeloid population, although appear to localize away from with microglial cells.

As we also previously observed differences in the clustering behavior of TAM-Supp cells between edge and core regions (Fig. 2F), we subsequently calculated the proportion of TAM-Supp cells that had infiltrated into the tissue in the edge and core regions of the tumor. This showed that significantly more TAM-Supp cells had infiltrated into the tissue in the core, suggesting that the vasculature may be a point of entry for TAM-Supp cells, which remain primarily associated with the vasculature in edge regions but infiltrate into the tumor in the core (Fig. 3F).

Overall, myeloid cells showed a propensity to have less interactions than would be expected by chance with tumor and neuroglial cells to an extent that was broadly similar between different myeloid populations. Observations were also similar between edge and core other than neuroglial cells having reduced impact on myeloid positioning in core regions. This suggests that differences in localization of specific myeloid populations are not strongly driven by the avoidance of (or preference for) direct heterotypic cell-to-cell interactions with neuroglial or tumor cells. The observed colocalization of macrophages with tumor cells may therefore be driven by common factors or tissue signals that do not rely on cell-cell interactions. By comparison, there was a clear population-specific association of TAM-Supp cells with the vasculature.

### Macrophages preferentially localize to hypoxic areas of the TME in GBM

**Fig. 4.**
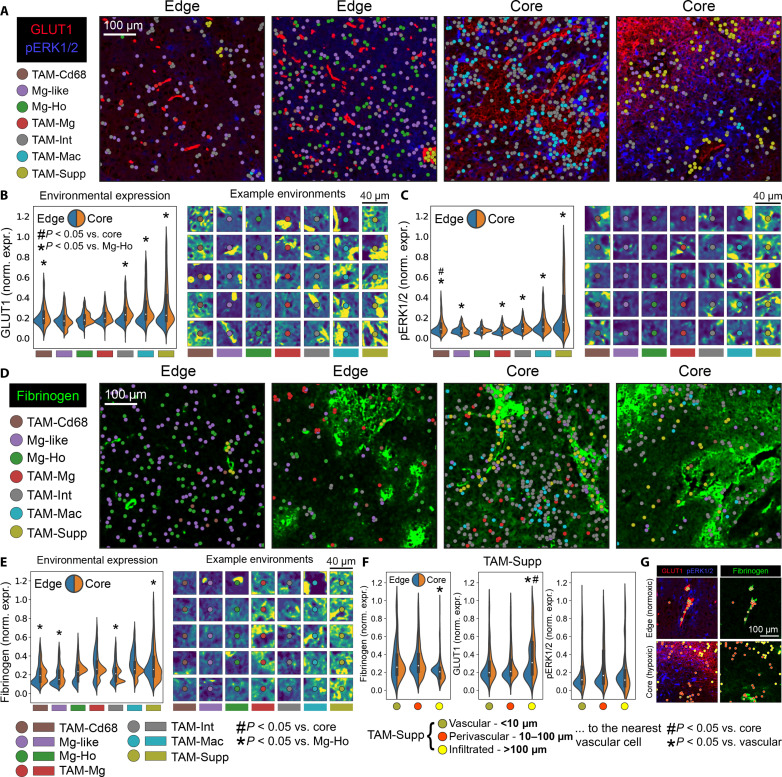
Association of hypoxia and fibrinogen with myeloid cell positioning in GBM. Myeloid populations mapped alongside markers of (**A**) environmental hypoxia in GLUT1 and pERK1/2 staining, or (**D**) fibrinogen. Quantification of environmental GLUT1 (**B**), pERK1/2 (**C**), and fibrinogen (**E**) staining around each myeloid population in edge and core regions. A cell’s environment was defined as a 40-μm square centered on the cell. (**F**) Comparison of environmental stains in TAM-Supp cells that were either vascular associated, perivascular, or fully infiltrated into the tumor, in both edge and core regions. (**G**) Representative examples of TAM-Supp cells with different vascular associations in a normoxic edge region and a hypoxic core region. Comparisons made by linear mixed models with Holm-Šídák corrections (B, C, E, and F). Violin plots show range, interquartile range, and median of data (B, C, E, and F).

Previous analyses suggested that cell-intrinsic behaviors associated with specific myeloid cell populations, or common to all GBM-associated myeloid populations, are at least partly responsible for myeloid positioning in the TME. However, environmental factors and biological niches likely also affect myeloid cell activities and positioning. Tissue hypoxia, often leading to necrosis, is a defining feature of GBM. We therefore hypothesized that compartmentalization of different myeloid cell populations may be driven by the degree of hypoxia in the surrounding tissue, assessed here through the up-regulation of GLUT1 and pERK (phospho-Erk1/2, Thr202/Tyr204), markers which have been previously shown to increase in response to tissue hypoxia in GBM (*34*–*37*). Within the regions analyzed, surrogate markers of hypoxia (GLUT1 and pERK) were not uniformly distributed, suggestive of discrete hypoxic niches within the tumor (Fig. 4A). Specifically, GLUT1 was constrained to the vasculature in edge cases but was found more diffusely in the parenchyma in the core in a pattern which has previously been observed in GBM and is thought to represent metabolic adaptation to tissue hypoxia (*35*). We therefore measured the GLUT1 and pERK staining in the local environment surrounding each myeloid cell (Fig. 4, B and C). This demonstrated that macrophage populations (TAM-Mac and TAM-Supp) were in environments with significantly higher expression of GLUT1 and pERK than microglia, with expression being highest in the TAM-Supp population. This suggests that macrophages preferentially localize to areas of hypoxia, while microglia are typically found in comparatively normoxic areas. However, vascular-associated GLUT1 may also be contributing to the association of TAM-Supp cells with GLUT1, as we have previously established that they cluster in vascular locations (Fig. 3). Similar analyses were also performed for CD8^+^ T cells and HIF1a^+^ immune cells (fig. S4), showing that the HIF1a^+^ cell population also preferentially localized to areas of hypoxia. CD8^+^ T cells positioned in areas with enriched environmental staining of pERK and within areas that trend higher for GLUT1, which is suggestive that they also localize to hypoxic areas.

A further environmental factor and feature of GBM pathology is the breakdown of the blood-brain barrier (BBB), which is associated with inflammation and aberrant angiogenesis (*14*). We therefore identified areas of BBB damage in both the edge and core of the tumor by measuring the presence of fibrinogen in the tumor, which was restricted to the lumen in vessels with an intact BBB but which leaked into the brain when the BBB was compromised (Fig. 4D). When we quantified the environmental localization of fibrinogen around each cell, we found that TAM-Supp cells localized to areas with significantly higher fibrinogen than microglia (Fig. 4E). This suggests that TAM-Supp cells localize to areas of BBB breakdown. However, as we previously established that TAM-Supp cells are vascular associated, fibrinogen trapped in intact vessels could contribute to this environmental enrichment. We therefore repeated previous analyses but separated TAM-Supp cells into vascular, perivascular, or fully infiltrated. This demonstrated that TAM-Supp cells in vascular and perivascular locations had similar environmental localization of markers but that cells that had fully infiltrated had significantly lower fibrinogen but higher GLUT1 association (Fig. 4, F and G). Although most TAM-Supp cells were vascular associated (Fig. 3), these results suggest that infiltrative TAM-Supp cells localize in areas of hypoxia and thus that hypoxia may be a signal that draws them away from their usual vascular and perivascular locations.

### Myeloid populations identified by IMC in GBM align with those defined through single-cell RNA sequencing

**Fig. 5.**
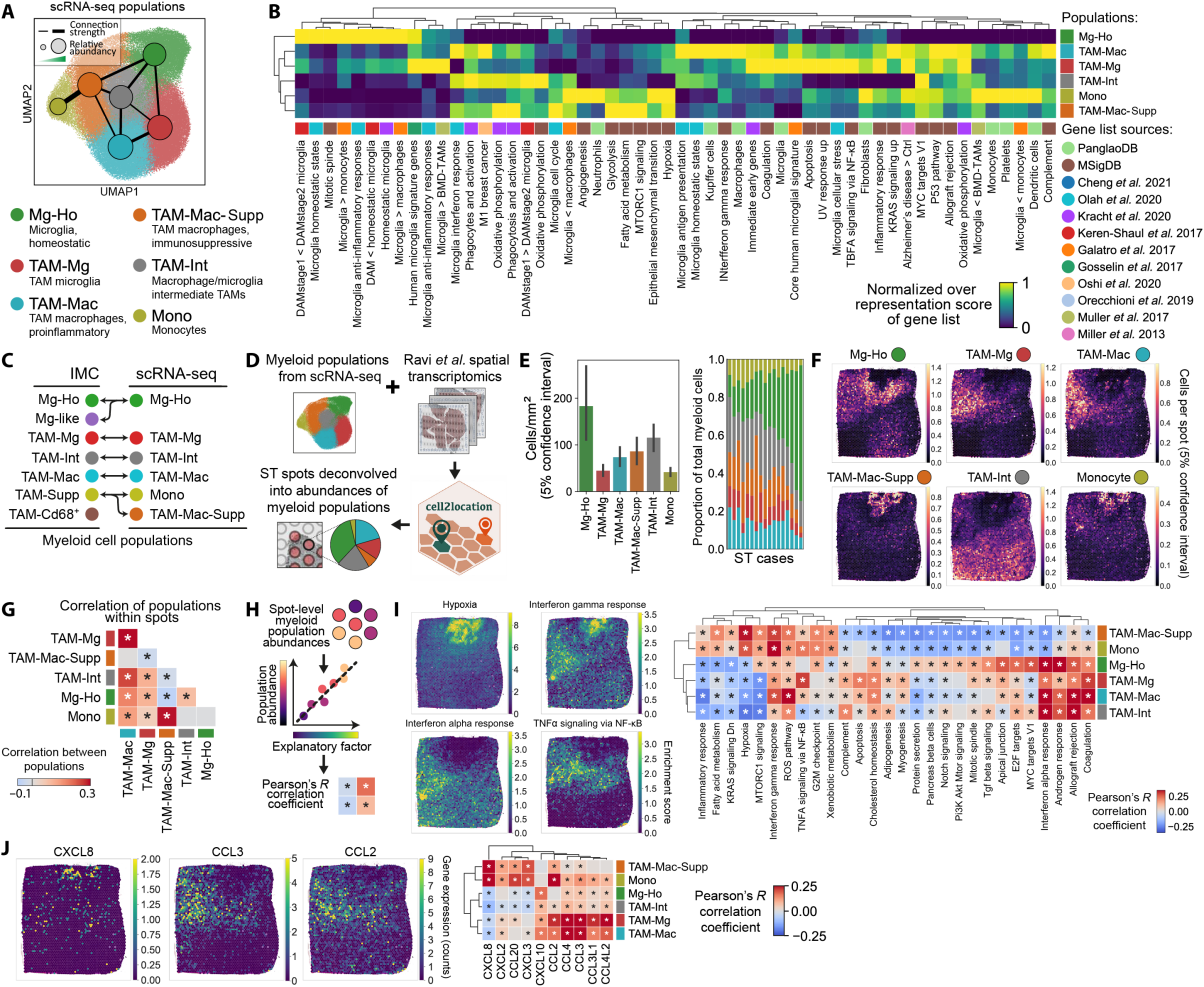
ST reveals hypoxia and chemokines as determinants of myeloid cell positioning in GBM. (**A**) UMAP of the myeloid populations identified by Leiden clustering in the Ruiz-Moreno *et al.* scRNA-seq dataset. (**B**) Comparison between the transcriptomes of the myeloid populations and published gene lists for biological processes (MSigDB) (*79*), cell identities (PanglaoDB) (*80*), myeloid cell phenotype in GBM, and other conditions disease (*7*, *70*–*78*). Enrichment of the gene lists was calculated by overrepresentation analysis. (**C**) Alignment of the populations identified by IMC and scRNA-seq. (**D**) Schematic showing the strategy for deconvolving the Ravi *et al.* (*37*) ST datasets using the cell2location (*82*) and the transcriptomic signatures of the myeloid populations we identified in the Ruiz-Moreno *et al.* dataset (*26*). (**E**) Predicted abundances of the myeloid populations calculated using cell2location. Data shown as mean ± SEM. (**F**) Distribution of myeloid populations in ST spots within a representative deconvolved ST case. (**G**) Pearson’s *R* correlation between the different myeloid populations present in each spot. (**H**) Strategy for identifying explanatory factors that may control the positioning of myeloid cell populations. Using this strategy, myeloid cell abundances were correlated with the transcriptomic signatures of biological processes from the MSigDB database (**I**) or expression of chemokines (**J**). *P < 0.05, with correction for multiple Pearson’s *R* tests using the Benjamini-Hochberg procedure (G, I, and J). UV, ultraviolet; NF-κB, nuclear factor κB; TNFα, tumor necrosis factor–α; ROS, reactive oxygen species.

We next took an orthogonal approach to validate the environmental drivers of myeloid cell positioning in the GBM TME. We characterized the myeloid cell populations present in GBM tumors by reanalyzing a published single-cell RNA sequencing (scRNA-seq) dataset (*26*). This identified six populations, including two populations of microglia (Mg-Ho; homeostatic microglia, and TAM-Mg; proinflammatory microglia), two of macrophage-derived TAMs (TAM-Mac and TAM-Mac-Supp), a population of TAM-microglial intermediate cells (having features of both microglia and macrophages), and monocytes (Fig. 5, A and B, and fig. S5A). Clear differences were found in inflammatory, metabolic, and proliferative signaling between these populations (fig. S5, B and C). There was a similar gene expression distribution of the markers utilized in IMC across the myeloid populations identified in the scRNA-seq dataset, as was observed at a protein level for the populations identified by IMC, indicating consistency in identified myeloid populations between modalities (Fig. 5C and fig. S5D). In some cases, populations found as two populations in one modality were represented as one in the other modality. For example, monocytes were not separable from macrophage-derived TAM populations in IMC but were distinguishable by scRNA-seq. Overall, these analyses allowed us to validate populations identified by IMC and align them with their transcriptomic identities (Fig. 5C).

We then used the transcriptomic identities of the myeloid populations to deconvolve an ST dataset of 19 GBM cases taken from the tumor core (Fig. 5A), allowing us to predict the abundances for each population in each case (Fig. 5, D and E). Spatially mapping the deconvolved populations to individual spots demonstrated compartmentalization of populations, with clear segregation of where populations were found at their highest densities (Fig. 5F). Where different populations were found in the same spot, positive correlations were found between populations with similar phenotypes (e.g., Mono and TAM-Mac-Supp) and negative correlations between dissimilar populations (e.g., Mg-Ho with TAM-Mac-Supp) (Fig. 5G), supporting earlier observations made in IMC of phenotypically similar populations spatially associating (Fig. 2). Spot-level correlations between myeloid and nonmyeloid populations (including those identified within earlier IMC analyses) used in deconvolution were also found (fig. S6A). For example, TAM-Mac-Supp and Mono populations correlated positively with tumor cells but negatively with neuroglial populations. This is in agreement with earlier observations showing more interactions between tumor cells and myeloid populations, largely based on their high abundances in the tumor core (Fig. 1F and fig. S3C). Notably, these results do not correct for abundances of populations and operate at a larger scale (spots are 50 μm in diameter) than the cell-cell interaction analyses performed in the IMC analyses (Fig. 3E).

To understand the potential drivers of myeloid positioning, we correlated the abundance of the myeloid cell populations with gene signatures of biological processes within each spot (Fig. 5, H and I). This confirmed findings made by IMC, with TAM-Mac-Supp and Mono populations preferentially accumulating in areas of increased hypoxic signaling and altered metabolism, which were associated with GLUT1 gene (*SLC2A1*) expression (fig. S5B). *HIF1A* expression was increased in surrounding areas of the tumor, although not where the hypoxic signature was highest. This is in agreement with recent studies that found *HIF1A* localized at the edges of areas with the highest hypoxia in a mouse model of GBM (*38*), and a second used a gene signature to spatially map reactive hypoxic niches in GBM that did not include *HIF1A* (*37*). This is suggestive of differential degrees of hypoxia throughout the TME, as has previously been reported (*39*). By contrast, the remaining myeloid populations were positively associated with signatures of interferon-α, androgen, and coagulation responses. Proinflammatory signaling pathways (interferon-γ, tumor necrosis factor–α, and reactive oxygen species) also influenced the positioning of specific myeloid subpopulations (Fig. 5I). Gene signatures of specific cytokine signaling pathways also correlated with myeloid abundances within spots (fig. S6C). As has recently been reported (*38*), the strength of interleukin-1B (IL-1B) signaling correlated positively with abundances of TAM-Mac-Supp and Mono populations. Repeating this analysis for chemokine genes suggested that the positioning of specific subsets of myeloid cells was also controlled by distinct groups of chemokines (Fig. 5J and fig. S6D). The same myeloid populations responsive to hypoxia (TAM-Mac-Supp and Mono) associated with *CXCL8*, *CXCL2*, *CCL20*, and *CXCL3*, whereas TAM-Mg and TAM-Mac populations were associated with *CCL4*, *CCL4*, *CCL3L1*, and *CCL4L2*. By contrast, the positioning of Mg-Ho and TAM-Int were only weakly associated with chemokine expression. Other chemokines were less robust differentiators of myeloid cell positioning, and in contrast to recent observations (*40*), we did not find close association of *CCL8* with hypoxic regions (Fig. 5J and fig. S6D). Together, these analyses reinforce and expand on findings made by IMC, showing that specific myeloid populations accumulate in hypoxic niches in the TME, and suggest a role of spatially variable chemokine and inflammatory signaling in myeloid compartmentalization.

### Myeloid cell environments were defined by distinct patterns of myeloid populations within ST spots

**Fig. 6.**
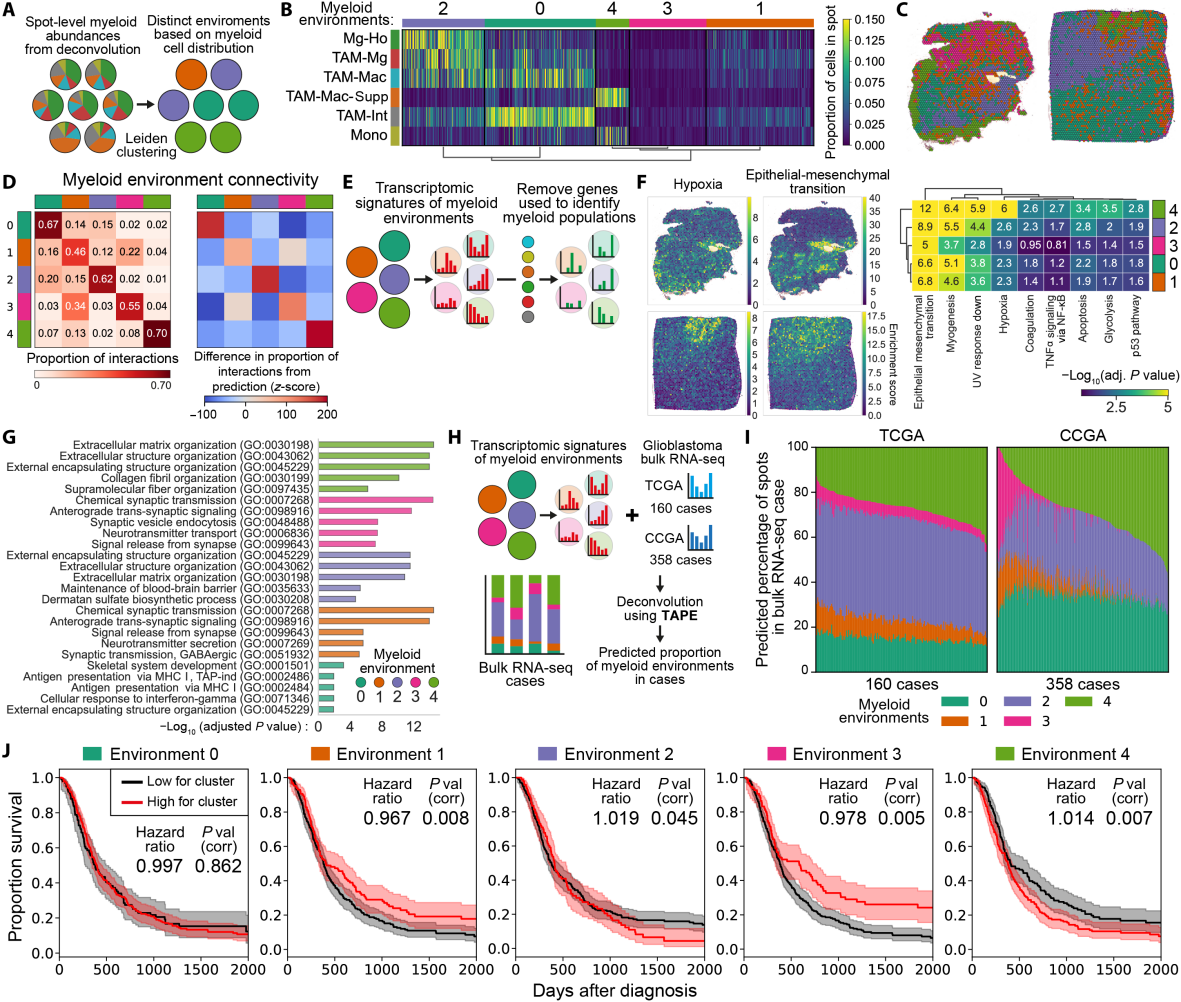
Spatial clustering of myeloid cells is associated with poor outcome in GBM. (**A**) Strategy to identify environments in the deconvolved ST cases that have distinct patterns of myeloid cell distribution. (**B**) Identification of distinct patterns of myeloid cell distribution using *k*-means clustering, corresponding to five distinct myeloid cell environments. (**C**) Mapping of the five myeloid environments in example ST cases. (**D**) Proportion of interactions between spots from each myeloid environment, assuming that each spot is connected to its neighboring six spots. (**E**) Strategy to identify the transcriptomic changes arising from the nonmyeloid cells in each spot (see Materials and Methods). (**F**) Overrepresentation analysis of the signatures of biological process from the MSigDB database (*79*) in the remaining nonmyeloid genes in the different myeloid environments. (**G**) The top five enriched terms from the Gene Ontology (GO) database in the nonmyeloid genes in the different myeloid environments. (**H**) Strategy to deconvolve bulk RNA-seq GBM cases from the TCGA (The Cancer Genome Atlas) and CCGA (Chinese Glioma Genome Atlas) using the TAPE algorithm (*41*–*43*), therefore allowing us to predict the proportions of myeloid environments in each case. (**I**) Proportion of myeloid environments predicted by the TAPE algorithm in the TCGA and CCGA cases. (**J**) Modeling the relationship between the abundance of each myeloid environment and GBM survival using Cox proportional hazards, with correction for multiple tests using Holm-Šídák. Hazard ratios are increase in risk of death per percentage point of myeloid environment abundance. Kaplan-Meier curves compare patients having the high (top 50%) or low estimates for the presence of that environment, and shaded areas are 95% confidence intervals.

Both IMC and ST analyses suggested that specific myeloid niches exist in the GBM TME, characterized by common biological processes (e.g., hypoxia), and occupied by myeloid populations with similar phenotypes. To identify these myeloid niches (hereafter termed myeloid environments), we clustered ST spots based on their abundances of the six myeloid populations (Fig. 6A). This identified five distinct myeloid environments, including three in which myeloid cells were highly abundant (0, 2, and 4) but in different combinations of phenotypically similar populations and two with low abundance (1 and 3) of myeloid cells (Fig. 6, B and C). This supports observations made in IMC whereby if cells were clustered, then it was with cells of the same or similar phenotype. Assessing the distribution of these environments within the TME found that they organize into larger regions spanning several interconnected spots, with the proportion of connections between spots being therefore dominated by those between spots of the same environment (Fig. 6D).

### Contribution of the nonmyeloid components to the myeloid environments

We then analyzed how the nonmyeloid components (i.e., contribution of genes from nonmyeloid cells) varied between the different myeloid environments, specifically focusing on genes not differentially expressed between myeloid populations (Fig. 6E). In these nonmyeloid components, there was a clear signature of hypoxia and metabolism in environment 4 (Fig. 6F), an environment enriched for myeloid populations which individually correlated with the signature of hypoxia (Fig. 5I). This supports the existence of hypoxic niches in the tissue, also observed by IMC (Fig. 4), which affects the behavior and positioning of both myeloid and tumor cells. In all five environments, there were processes likely arising from the tumor cells (e.g., epithelial-mesenchymal transition, p53 pathway). This support observations made by IMC (Fig. 3 and fig. S3) and deconvolution of ST populations (fig. S6A) that nonmyeloid cells influence the positioning of the myeloid cell populations. To obtain further insight into the nonmyeloid factors which differentiate the myeloid environments, we performed Gene Ontology on the nonmyeloid differentially expressed genes (DEGs) between myeloid environments (fig. S7A and Fig. 6G). This demonstrated that environments 2 and 4 each had unique differences in extracellular matrix composition. Given these two environments were enriched for phenotypically different myeloid cells, it suggests that myeloid cell compartmentalization may be influenced by the local deposition of extracellular matrix components by nonmyeloid cells. The two environments with lowest abundance for myeloid cells (1 and 3) were enriched for genes associated with neuronal functioning, suggesting that neural progenitor–like tumor cells may influence myeloid positioning by inhibiting local myeloid recruitment.

### The transcriptomic signature of specific myeloid environments was associated with reduced disease survival

The complete transcriptomic signatures of the myeloid environments were then used to deconvolve 518 bulk mRNA sequencing GBM cases from The Cancer Genome Atlas (TCGA) and Chinese Glioma Genome Atlas (CCGA) datasets using the TAPE (Tissue-AdaPtive autoEncoder) algorithm (Fig. 6H) (*41**–**43*). This analysis allowed us to predict the proportions of myeloid environments present in each case, showing clear variability between patients, particularly in environment 4 which is dominated by TAM-Mac-Supp and monocytes (Fig. 6I). Comparing the overall survival curves for patients either low or high for each myeloid environment (either lower or higher than the mean) and performing univariate COX proportional hazard analyses showed that the abundance of the myeloid environments has a significant effect on GBM survival. Specifically, tumors with high proportions of environments 2 (featuring clustering of proinflammatory populations) and 4 (featuring clustering of immunosuppressive populations) were associated with worse survival, whereas high proportions of environments 1 and 3 (with low myeloid clustering) were associated with better survival. As the proportions were correlated with one another, a multivariate model with the five environments could not be built. However, a multivariate model using principal components of the five environments also showed a significant effect of varying myeloid abundance on survival (fig. S7B). Together, these IMC and ST analyses demonstrate that the spatially regulated compartmentalization of myeloid cell populations, which is instructed through tumor environmental cues, contributes to disease trajectory during GBM.

## DISCUSSION

In this study, we have revealed the compartmentalization of key myeloid cell populations within GBM. Utilizing orthogonal high dimensional IMC analyses and deconvolved ST datasets, we robustly identified at least six myeloid cell populations. Microglia existed on a spectrum of activation states from homeostatic to proinflammatory activation, with the transition being associated with a reduction of P2RY12, supporting previous observations made in GBM (*7*, *22*, *29*). Macrophages were either immunosuppressive, showing up-regulation of markers previously associated with an immunosuppressive phenotype in GBM myeloid cells (CD163 and CD206) (*11*, *18*, *22*, *23*), or proinflammatory. In IMC analyses, these proinflammatory macrophages were distinguishable from proinflammatory microglia by further reduction in P2RY12 expression and up-regulation of CD14 and CD16. Furthermore, transcriptomic analysis demonstrated that they had increased interferon-γ and complement signaling. Although we could identify these myeloid states as distinct populations, there was usually a gradient change in marker expression between populations, which suggested that cells were transitioning between phenotypes. Specifically, diffusion pseudotime analysis suggested that microglia undergo proinflammatory activation and adopt a more macrophage-like phenotype, before lastly becoming immunosuppressive. This is in line with previous studies in GBM, which report myeloid cells in transitionary states between more obviously identifiable proinflammatory or suppressive phenotypes (*4*, *24*, *25*), with cells able to simultaneously coexpress M1 and M2 markers (*7*). Consequently, in both IMC and sequencing datasets, we found canonical strongly polarized TAM and microglial populations, as well as large intermediate populations that exhibited characteristics of microglia and macrophages and of mixed pro- and anti-inflammatory polarization. Notably, we could not distinguish monocytes from other myeloid populations using the IMC panel used in this study. We acknowledge this limitation and that the established route of infiltrating monocyte tumor–associated macrophage differentiation within the GBM TME will operate in parallel to the route of microglial differentiation we describe in this study (*17*, *18*, *22*). Nevertheless, although we were not able to directly address the factors that control infiltration and differentiation spatially within the GBM TME by IMC, we could see evidence of monocyte to macrophage differentiation in the scRNA-seq data and spatially mapped monocytes in the ST cases appeared to infiltrate and differentiate within hypoxic zones.

Our IMC analyses demonstrated that microglial cells tended to localize within the GBM tumor edge and macrophage-derived TAMs to accumulate in the tumor core, supporting previous observations made in both human GBM and in murine models (*11*, *16*, *24*, *28*, *44*, *45*). However, this compartmentalization was not mutually exclusive, as the numerous myeloid populations were present in each tumor region analyzed. However, the positioning of the different myeloid cells within the TME was nonrandom: Myeloid cells preferentially positioned with cells of the same or similar phenotype and positioned away from myeloid cells of a dissimilar phenotype. This could suggest some degree of active avoidance or exclusion of myeloid cells with other nonmyeloid cells, but it more likely reflects cells responding to stronger migratory cues from other immune and myeloid cells. This behavior differed in strength between populations, being strongest in immunosuppressive TAMs, and was also recapitulated when analyzing ST datasets. Furthermore, most cell-to-cell interactions made by myeloid cells were with other myeloid cells. These analyses suggest myeloid cell interactions as a major cellular determinant of myeloid cell positioning, which is not unexpected given their role as the primary producers of chemokines and other signals that elicit responses from myeloid cells (*5*). We found clear regionalization in chemokine genes and cytokine signaling pathways that correlated with myeloid cell positioning. Notably, although the abundance of certain myeloid populations differed between the edge and core regions of the tumor, the overall compartmentalization behavior of myeloid populations was broadly similar in both parts of the tumor. This suggests that the factors that coordinate myeloid cell activities (whether deriving from myeloid or tumor cells or the environment) are conserved throughout the GBM TME.

Hypoxia was identified as a major controller of myeloid cell compartmentalization within GBM, particularly influencing immunosuppressive populations. In IMC analyses, immunosuppressive myeloid cells (CD163^+^ and CD206^+^) were found to be most strongly linked to hypoxic areas, although this association was also observed, albeit less significantly, in other macrophage populations. In contrast, nonactivated microglia exhibited the lowest association with hypoxia across all analyses. This is in support of previous studies that have reported that macrophages, rather than microglia, accumulate in hypoxic areas of the TME (*9*, *22*). Immunosuppressive myeloid cells also showed an interaction with the vasculature in IMC analyses that was consistently observed throughout the TME. Further examination of this population by transcriptomics revealed that it was a combination of monocytes and immunosuppressive macrophages, explaining their vascular association. In agreement with IMC analyses, both monocytes and immunosuppressive macrophages exhibited a preferential localization within hypoxic niches in ST analyses. However, the presence of hypoxic signaling within the monocytes implied that most were already being influenced by the GBM environment and were likely differentiating into macrophages. We saw greater infiltration of immunosuppressive myeloid cells in IMC analyses into hypoxic areas of the tumor core. These observations are supported by previous characterizations of immunosuppressive myeloid cells in GBM, which have typically found them to be blood-derived macrophages with high expression of hypoxia-related genes (*9*, *17*, *18*, *28*). Our results are supported by recent studies that found similar localization of immunosuppressive myeloid cells, along with CD8^+^ T cells, to hypoxic zones within the TME in GBM (*37*, *38*). Together, our findings from IMC, ST, and scRNA-seq all indicate that hypoxia plays a pivotal role in the differentiation of myeloid populations in GBM toward an immunosuppressive phenotype, as reported in TAMs in various cancer types (*37*, *38*, *46*). Nevertheless, as acute hypoxia in the context of stroke leads to polarization of proinflammatory myeloid cells (*47*), it is likely the prolonged exposure to hypoxia in context with tumor-specific factors that collectively promotes the polarization of myeloid cells to the immunosuppressive phenotype within the GBM TME.

Accumulation of myeloid cells in hypoxic niches could also be attributed to the release of chemotactic signals induced by hypoxia (*46*). Our investigation unveiled heterogeneous expression of chemokines throughout the TME, with specific chemokines correlating with immunosuppressive populations in hypoxic regions. Other chemokines (e.g., *CCL-2*, *CCL-3*, and *CCL-4*) were associated with the positioning of proinflammatory macrophage and microglial populations. This in agreement with previous studies reporting similar populations located at the tumor periphery serve as the primary source of these chemokines, with authors hypothesizing that they are responsible for recruiting additional myeloid cells (*5*, *28*, *44*). Alternatively, these chemokines may themselves affect myeloid phenotype, with CCR2 knockout resulting in a greater proportion of microglia in mouse tumors, although not by blocking monocyte infiltration but by blocking monocyte-to-macrophage differentiation (*22*). In agreement with a recent report (*38*), IL-1B signaling was spatially associated with immunosuppressive myeloid cells in hypoxic zones, although we could not confirm a similar association of *CCL8* due to poor detection of *CCL8* in the ST cases.

It is also highly probable that myeloid compartmentalization is influenced by the positioning and interaction with tumor cells. However, compared to some solid tumors, the different components of the TME in GBM do not commonly show a macroscopically obvious segregation into defined immune and tumor compartments, likely due to the highly infiltrative nature of tumor cells in GBM. Unexpectedly, we found that all myeloid populations showed some degree of spatial avoidance (i.e., a preference to localize away from) of tumor and neuroglial cells in the TME. Although this may represent an activate avoidance of tumor cells by myeloid cells, as discussed above, myeloid cells likely preferentially respond to other cues from other myeloid cells that dictate their positioning. A potential caveat of our analyses is that we did not differentiate between the three known neoplastic subtypes present in GBM (*27*, *48*). However, existing data suggest that they may differentially shape the myeloid landscapes in GBM. For example, GBM tumors rich in mesenchymal subtype have the highest myeloid cell density, while those abundant in proneural subtype show the lowest macrophage proportion (*6*, *49*). Myeloid cells can directly shape tumor cell fate, with macrophages being shown to drive differentiation of tumor cells toward a mesenchymal phenotype (*50*). Further studies are therefore required to address how specific myeloid and neoplastic cell subtypes interact during GBM.

The clinical significance of the spatial arrangement of immune cells within a tumor has been demonstrated in various cancer types (*23*, *40*, *51*), frequently offering superior prognostic value compared to the mere abundance of immune cells (*52*–*54*). Because of the previously described clustering of myeloid populations in different areas of the TME, we were able to extract the transcriptomic signatures of the different spatial arrangements of myeloid populations. These different myeloid environments were each dominated by myeloid cells of a different phenotype. When we then deconvolved 518 bulk RNA-seq cases using these signatures, we found reduced survival time in patients with tumors that were enriched with environments where either proinflammatory or immunosuppressive populations were highly clustered. This demonstrates that the topology of myeloid populations in GBM is associated with disease outcome. Specific immunosuppressive myeloid populations identified using scRNA-seq (e.g., *CD73* and *MARCO* high) have been associated with poor survival in GBM (*9*, *10*). Our spatial analyses add context to these findings, suggesting that these cells are located in hypoxic areas and are likely vascular-associated. Proinflammatory macrophages are associated with disease progression in lower-grade gliomas, with fewer immunosuppressive macrophages compared to GBM (*55*–*57*). The detrimental effect of proinflammatory niches may therefore represent areas transforming from low- to high-grade tumor. Previous studies have demonstrated that tissue hypoxia detected by magnetic resonance imaging (*39*, *58*) or by hypoxic-responsive factors (*35*, *59*) is an indicator of poor prognosis in GBM. Our results add spatial context to these findings, suggesting that an important component through which hypoxia controls progression and treatment responsiveness is by shaping the positioning and phenotype of myeloid cells. Overall, these results align with an expanding body of research that indicates that the spatial structure of the GBM TME plays a decisive role in determining the clinical course (*23*, *60*). Ultimately, better understanding of the biology of GBM and revealing how cells communicate within the TME will allow us to start deconstructing the heterogeneity in GBM and stratify patients for targeted treatments.

## MATERIALS AND METHODS

### Clinical samples for IMC

Eight primary IDH^WT^ GBM cases were retrieved from the Department of Cellular Pathology at Salford Royal Hospital bank [Table 1; Ethics Integrated Research Application System (IRAS) ID 244538 for informed consent of tissue use in research]. The interface between tumor and cortex (edge) and the tumor core were annotated by a neuropathologist on H&E-stained sections. TMAs were subsequently generated using 3-mm-diameter cores, with three cores taken per case.

### IMC tissue staining

Sections from TMAs (5-μm thickness) underwent staining with lanthanide-conjugated antibodies as instructed by the manufacturer (*61*). Briefly, sections underwent deparaffinization, followed by antigen retrieval at 96°C for 30 min in tris-EDTA at pH 8.5. Nonspecific binding was blocked with 3% bovine serum albumin for 45 min, followed by incubation with lanthanide-conjugated primary antibodies (overnight at 4°C) which were diluted in phosphate-buffered saline (PBS) with 0.5% bovine serum albumin (Table 2). Antibodies were conjugated with metals using Maxpar Antibody Labeling Kits (Standard BioTools) and were validated with positive control tissue (tonsil for immune-targeted antibodies), and dilutions were optimized with GBM tissue. Slides were then washed with PBS and 0.1% Triton X-100 in PBS. Slides then underwent nuclear staining with iridium (1:400, Intercalator-Ir, Standard Bio Tools) for 30 min at room temperature, before being briefly (10 s) washed with ultrapure water and air-dried. Images were acquired of metal-stained tissue sections on a Hyperion IMC as per the manufacturer’s instructions (Standard BioTools). Each TMA core was imaged in a separate region of interest. Briefly, the tissue was laser-ablated in a rastered pattern in a series of 1-μm^2^ pixels. The resulting plume of ablated tissue was then passed through a plasma source, ionizing it completely into its constituent atoms. Time-of-flight mass spectrometry then discriminated the signal for each of the metal-conjugated antibodies, and images for each antibody were reconstructed based off the metal abundancy at each pixel. Staining was reviewed by a neuropathologist using MCD Viewer (Standard BioTools). In representative images of IMC, data shot noise was removed using the IMC-Denoise algorithm (*62*).

**Table 2.** Antibody panel for IMC.

Metal channel	Antigen	Type	Clone/catalog #	Supplier
89	Smooth muscle actin	Vascular	1A4	Abcam
113	Cd68	Myeloid	KP1	BioLegend
115	Cd235ab	Vascular/erythrocytes	KIR2	BioLegend
139	Pan-cytokeratin	Epithelial	AE-1/AE-3	BioLegend
141	S100B	Neoplastic	EP1576Y	Abcam
142	MHC1	Immune	EMR8–5	Abcam
143	Vimentin	Neoplastic	RV202	Standard BioTools
144	Cd14	Myeloid	D7A2T	Cell Signaling
145	Ki67	Proliferation	B56	BD Pharmingen
146	Cd16	Myeloid	SP175	Abcam
148	Cd66b	Myeloid	G10F5	Novus
149	Cd11b	Immune	EP1345Y	Abcam
150	Cd44	Neoplastic	IM7	BioLegend
151	Granzyme B	Immune	EPR20129–217	Abcam
152	Cd45	Immune	CD45-2B11	eBioscience
153	Cd31	Vascular	JC/70A	Novus
154	Cd11c	Myeloid	EP1347Y	Abcam
155	HIF1a	Signaling	EP12154	Abcam
156	Cd4	Immune	EPR6855	Abcam
158	Cd109	Neoplastic	C9	Santa Cruz
159	Olig2	Neoplastic	Polyclonal, AB9610	Millipore
160	Vista	Myeloid	D5L5T	Cell Signaling
161	iba1	Myeloid	Polyclonal, 019–19741	Wako
162	Cd8a	Immune	CD8/144B	eBioscience
163	GLUT1	Signaling	EPR3915	Abcam
164	Nestin	Neoplastic	25/Nestin	BioLegend
165	Fibrinogen	Vascular	EPR18145–84	Abcam
166	Cd74	Myeloid	LN2	BioLegend
167	Met	Signaling	Met	D1C2
168	P2RY12	Myeloid	HPA014518-100UL	Merck
169	Cd163	Myeloid	EDHu-1	Bio-Rad
170	Cd3	Immune	D7A6E	Cell Signaling
171	pERK1/2	Signaling	D13.14.4E	Cell Signaling
172	TMEM119	Myeloid	Polyclonal, ab185333	Abcam
173	Sox2	Neoplastic	245610	R&D
174	MHCII	Immune	TAL1B5	Abcam
175	Cd206	Myeloid	E2L9N	Cell Signaling
176	GFAP	Neoplastic/astrocytes	GA5	Sigma-Aldrich

### Cell segmentation of IMC images

Single-cell information was extracted from IMC images using an established protocol (*63*). Briefly, stacks of TIFF images were extracted from MCD files for each region of interest whereby individual channels corresponded to each lathanide-conjugated antibody. Ilastik (*64*) was then used to produce a pixel probability classifier that identified background, cytoplasmic, and nuclear pixels. The resulting pixel probability maps were then converted into cell segmentation masks that identified the regions corresponding to individual cell boundaries. These cell segmentation masks were then applied to each of the antibody channels, generating single-cell expression data for each of the channels, along with the spatial context of where the cell was in the tissue. The accuracy of cell segmentation was compared to manual segmentation in 50 random cells per TMA core by Jaccard analysis using Scikit-Image (v0.22.0), with the resulting accuracy being comparable to similar published approaches (*15*).

### Analysis of single-cell IMC data

Single-cell IMC data were analyzed in Python using packages designed to analyze single-cell data (Scanpy, v1.9.3) and spatial molecular data (Squidpy v1.2.3 and ATHENA v0.1.3) (*65*–*67*). The mean cell intensity of each marker was normalized to the 99th percentile of its expression. Leiden clustering (*19*) was then used to identify cell populations present in the IMC data, which were then manually annotated based on patterns of marker expression corresponding to known cell types and activation states. The transition between myeloid populations was assessed using diffusion pseudotime and PAGA analyses (*68*) using Scanpy.

### Spatial distribution and interaction analyses in IMC data

Metrics were calculated at the single-cell level, before being mean averaged at the population level for each region of interest. For spatial distribution analyses, the distance between each cell and the nearest member of each other population was calculated. For interaction analyses, the number of interactions made by each cell (either calculated as six nearest neighbors or cell-to-cell contact) to other populations was calculated and expressed as a proportion of total interactions. The observed values for mean distances and proportions of interactions were then subtracted from the values predicted by a random distribution of cells, which was calculated by randomly distributing cell labels 300 times within each region. This corrected interactions for differences between regions in abundances of interacting populations. The resulting differences between observed and predicted values were separately averaged across all edge and core regions, with statistical difference from random distribution (observed − predicted = 0) assessed using Wilcoxon tests with Benjamini-Hochberg correction. Cross-pair correlation analysis was also applied to quantify interactions between cells (*32*, *33*). This method measured whether cells from different populations were found within a 20-μm distance of each other (indicating cell-cell interactions) more or less frequently than what a random distribution of populations would predict. Rao’s quadratic entropy is a measure of phenotypic heterogeneity and was calculated between each cell and the cells it makes direct cell-to-cell contact using ATHENA (*67*), with values mean averaged at the population levels for each region.

### Clustering and assortativity measures of myeloid cells in IMC data

Clustering coefficients (*30*) and assortativity (*31*) were calculated on six–nearest neighbor graph of myeloid cells in each region using Networkx (v3.1). A high clustering coefficient that designates a population forms densely interconnected clusters, and a low value suggests that population is more loosely clustered in the TME (*30*). Assortativity is another measure of clustering in a network and measures the tendency of cells to connect to cells of the same population rather than cells of a different population (*31*). Clustering coefficients for each population were calculated by extracting each population as a subgraph and calculating their average clustering coefficients. The observed clustering coefficients were then compared to a random distribution as described above, whereby cell labels were randomly distributed 300 times.

### Cell environment analysis in IMC data

The environmental expression of GLUT1, pERK1/2, and fibrinogen was calculated by taking the mean average expression of each marker in a 40-μm-diameter window centered on each cell. The distribution of the resulting environments was then statistically compared between cells from either edge or core of the tumor or between different populations using linear mixed models (LMMs) in which cells were nested within regions, which were nested within individual cases. Correction for tests was performed using a Holk-Šídák correction.

### Reanalysis of myeloid cells from a single-cell sequencing dataset

We analyzed 127,339 myeloid cells from a multistudy scRNA-seq dataset that incorporated 240 patients from 26 separate sequencing studies (*26*). We analyzed a 25% random sample of cells labeled as microglia, macrophages, or monocytes by the original authors. Batch effects were corrected using the Harmony algorithm (*69*), populations were identified using Leiden clustering (*19*), and connectivity of the populations was assessed using PAGA analysis (*66*, *68*). Overexpression analyses were performed with the Decouplr (v1.3.4) using hypergeometric tests (false discovery rate < 0.05). These used published gene lists from studies assessing the phenotypes of microglial in other conditions (*70*–*75*), of myeloid cell activation (*76*), and myeloid cells in GBM (*7*) and other cancers (*77*, *78*). Gene lists from canonical pathways of biological processes provided by Molecular Signatures Database (MSigDB) (*79*) and of cellular identities from PanglaoDB (*80*) were also used. Activity inference for pathways from the PROGENy (Pathway RespOnsive GENes for activity inference) database (*81*) was performed using multivariate linear modeling.

### Deconvolution of ST dataset

We analyzed a published ST dataset of 19 IDH^WT^ GBM cases taken from the tumor core (*37*). Data analysis was performed using Scanpy. Briefly, low-quality spots (<1000 counts) and mitochondrial genes were removed, and counts were normalized per cell and log-transformed. The cell type composition of each spot was then calculated using cell2location (v0.1.3) (*82*). Reference expression signatures of the myeloid cell populations were created from the single-cell sequencing dataset by taking the mean over all cells within each population. Reference signatures for nonmyeloid populations were similarly created from the “annotation level 3” labels from the Ruiz-Moreno *et al.* dataset (*26*) and were included in the matrix of reference expression signatures to account for all potential cell populations present in the GBM TME, ensuring accurate deconvolution. The resulting abundances are the lower limit at which the model is confident, in other words, at least this amount is present.

### Predicting transcriptomic controllers of myeloid cell positioning

To understand factors that control myeloid cell positioning, the abundance of each myeloid cell population estimated from deconvolution was correlated with potential explanatory factors using Pearson’s *R* correlation. Factors that did not vary between populations [<0.05 standard deviation (STD)] were removed, and the remaining analyses were corrected for multiple tests using a Benjamini-Hochberg correction. Explanatory factors were either single genes (chemokines), signatures of hallmark biological processes provided by MSigDB (*79*), or gene signatures of responses to cytokine signaling provided by CytoSig (*83*), which were quantified in each spot using hypergeometric tests in Decouplr.

### Identification of myeloid environments and their transcriptomic signatures

To identify the different myeloid environments, spots were clustered on the basis of the estimated abundance of myeloid cells populations from deconvolution. Population data were scaled to unit variance and zero mean and batch-corrected between cases using the BBKNN (batch balanced nearest neighbours) algorithm (*84*). Distinct patterns of myeloid abundance (constituting different environments) were identified using Leiden clustering in Scanpy (*19*). For assessments of connectivity between environments, each spot was connected to each of its surrounding six spots. To investigate the contribution of nonmyeloid cells to the transcriptomes of the different environments, any genes with >0.1 (counts) STD between myeloid populations in the reference expression signatures used for deconvolution of ST data were excluded from analysis (2115 genes removed, leaving 10031). The remaining genes were then compared between environments using hypergeometric tests using gene lists from MSigDB and by calculating DEGs using Wilcoxon rank sum test (*85*). The resulting DEGs (*P* < 0.01, 1.5 fold enrichment, 117 genes per cluster) were then used for overrepresentation analysis by Enrichr web services via GSEApy (v1.0.4), accessing the Gene Ontology databases (*79*, *86*, *87*).

### Deconvolution of bulk sequencing

The transcriptomic signatures of the myeloid environments were used to deconvolve bulk mRNA sequenced from IDH^WT^ GBM cases from the TCGA PanCancer atlas (160 cases) and CCGA (358 databases) (*42*, *43*). Bulk mRNA data from both datasets were independently sequenced on the Illumina HiSeq V2 platform, count data RSEM (RNA-seq by expectation maximization) normalized, and batch normalized (*42*, *43*). Deconvolution to estimate the proportion of myeloid environments in each bulk case was then performed using the TAPE algorithm (*41*), which sampled 500 spots from each myeloid environment, and was ran with the following hyperparameters: variance threshold of 0.99, min-max scaling. The resulting proportions of myeloid environments were associated to patient survival using Cox proportional hazards models ran using the ehrapy (v0.3.0) (*88*). Kaplan-Meier curves compare patients having the high (top 50%) or low estimates for the presence of that environment.

### Statistical analyses

Statistical tests were performed using the statsmodels (v0.13.5) and ehrapy packages and are specified for individual methods. For cell environment analyses, calculations were at the cell level, and so for LMMs, both region and case were used as grouping factors. For all other LMMs, data were mean-averaged at the region level, and patient case was used a grouping factor. Where LMMs were used concurrently, a Holm-Šídák correction was used for the calculation of *P* values.
