# Supplementary material for: Hypoxia coordinates the spatial landscape of myeloid cells within glioblastoma to affect survival
Source: Sci Adv. 2024 May 17;10(20):eadj3301. doi: 10.1126/sciadv.adj3301 (PMC11100569; doi:10.1126/sciadv.adj3301)
Supplement: Supplementary file 1 — Figs. S1 to S7 [file sciadv.adj3301_sm.pdf]

Science Advances
AAAS

Supplementary Materials for

Hypoxia coordinates the spatial landscape of myeloid cells within glioblastoma to affect survival

Michael J. Haley *et al.*

Corresponding author: Kevin N. Couper, kevin.couper@manchester.ac.uk;
Michael J. Haley, michael.haley@manchester.ac.uk

Sci. Adv. **10**, eadj3301 (2024)
DOI: 10.1126/sciadv.adj3301

This PDF file includes:

Figs. S1 to S7

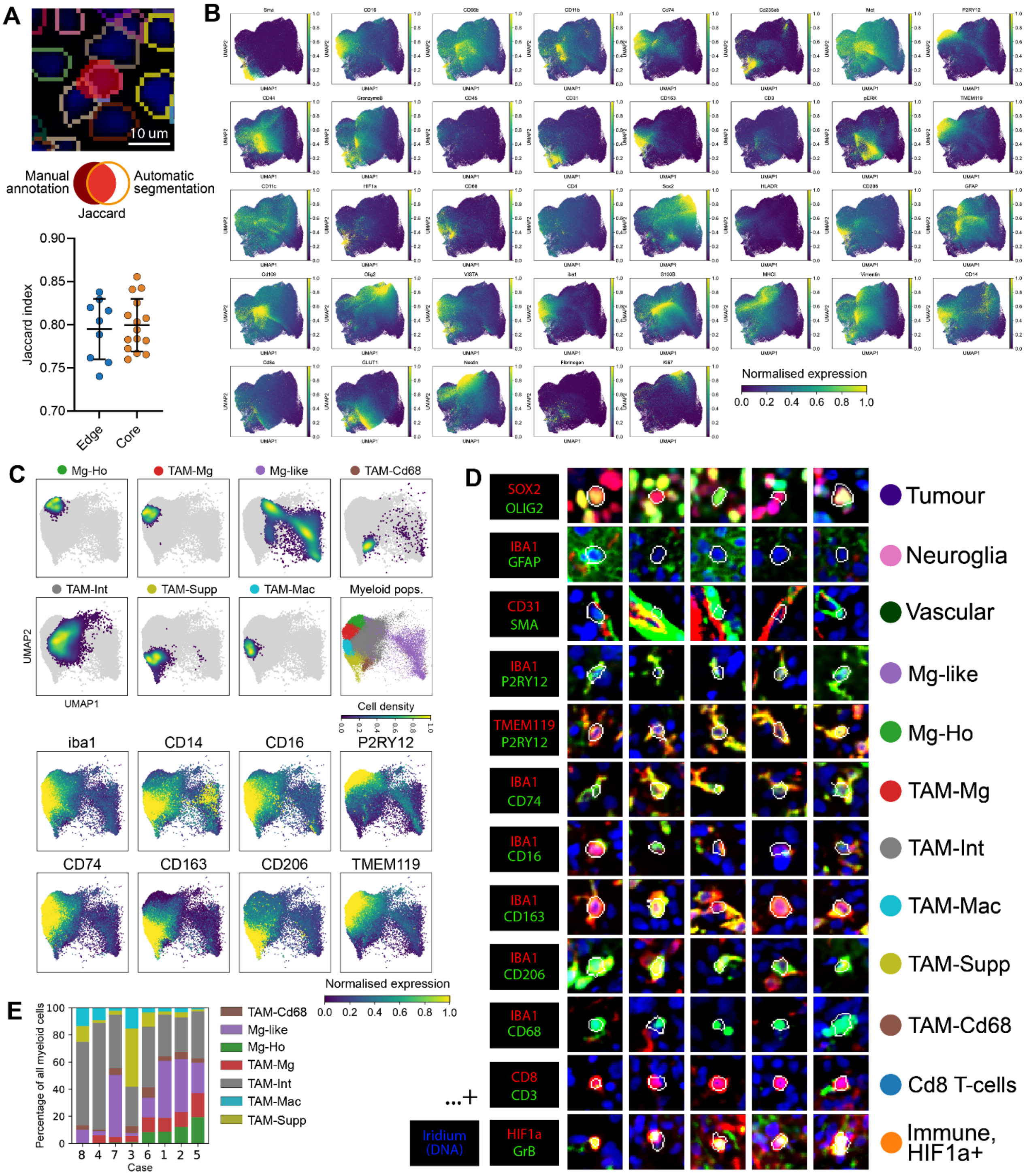

Supplemental fig. S1

A. Jaccard analysis demonstrating the overlap between manual annotation and the automatic segmentation pipeline. Data shown as mean ± standard deviation. **B**. UMAPs visualising the single-cell data acquired from the IMC workflow from all cases, demonstrating the distribution of all the markers in the panel. Each marker is normalised to the 99th percentile of its expression. **C**. UMAP embedding of the different myeloid populations alongside key myeloid markers used for population identification. **D.** Representative IMC images of each of the populations as identified and segmented (white border) in the tumour microenvironment. **E.** Percentage of all myeloid cells made up by the different populations in each case.

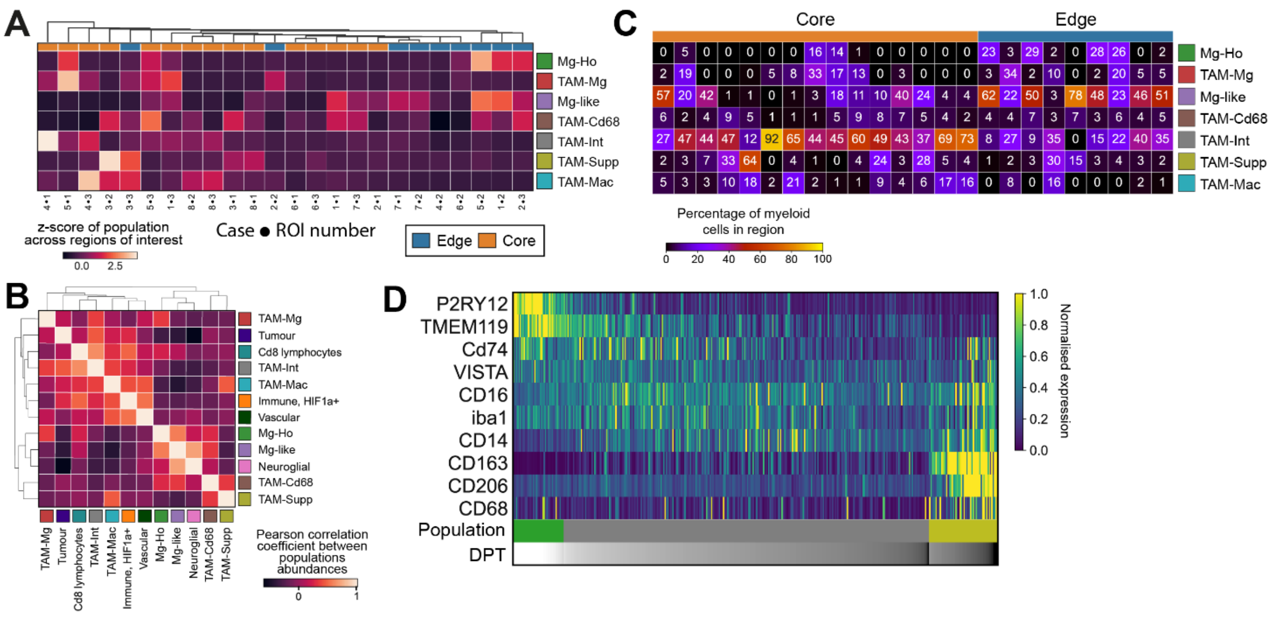

Supplemental fig. S2

A. *K*-means clustering of regions of interest based upon myeloid cell abundance. **B**. Pearson's correlation coefficient between populations in each region. **C**. Percentage of total myeloid cells made up by each population in each region. **D**. Diffusion pseudotime modelling a potential pathway of differentiation from homeostatic microglia (Mg-Ho), through a macrophage/microglia intermediary state (TAM-Int), into immunosuppressive myeloid cells (TAM-Supp).

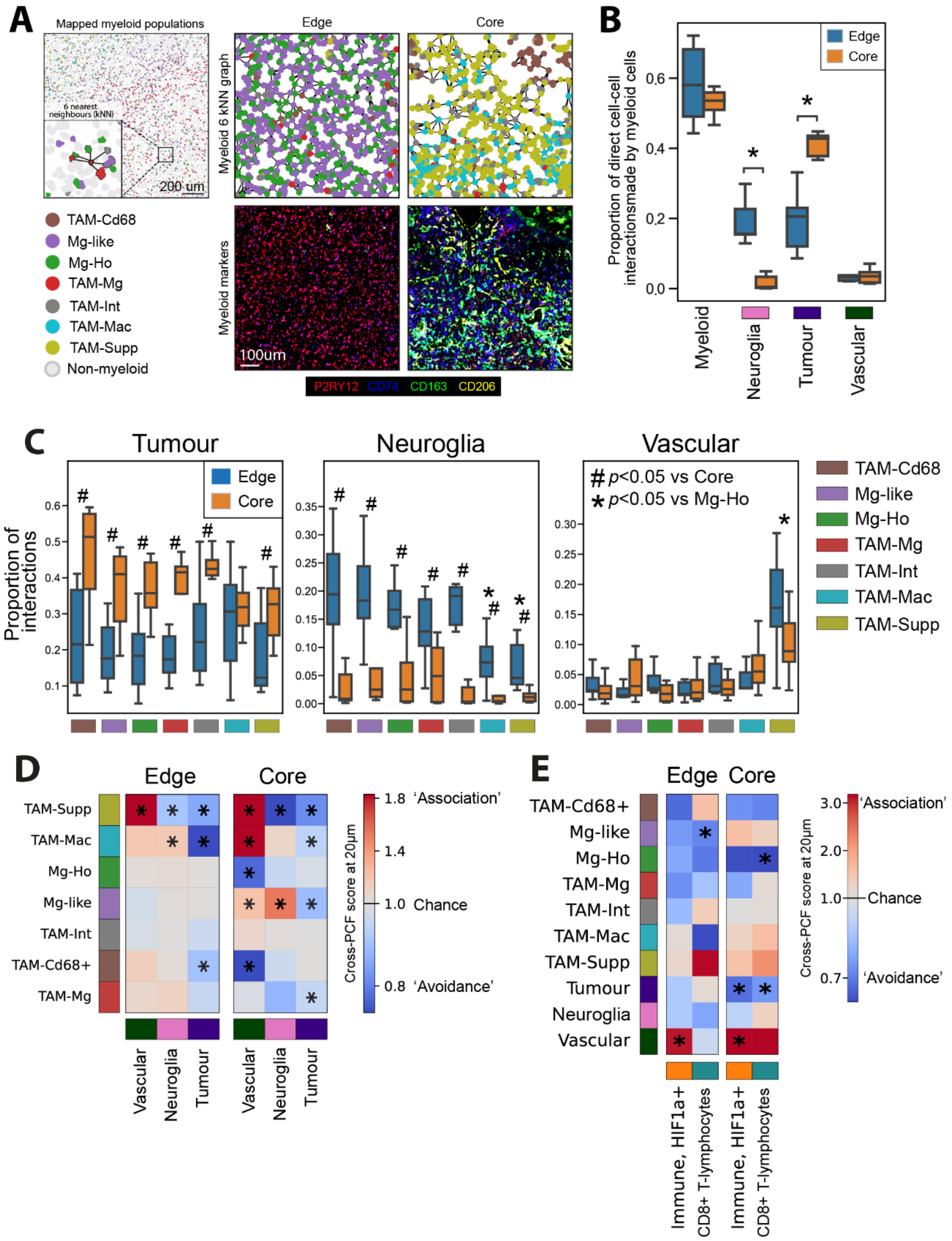

Supplemental fig. S3

A. Demonstration of how the distribution of myeloid cells can be assessed by connecting each myeloid cell to its nearest 6 neighbours, with representative examples of the resulting graphs from edge and core regions, and associated IMC images for myeloid markers. **B**. Proportion of cell-cell interactions made by all myeloid cells (not broken down into separate populations) with non-myeloid cells. Comparison made by linear mixed model. **C**. Proportion of direct cell-to-cell interactions made by myeloid populations with non-myeloid cells, compared between edge and core regions. Comparison made by linear mixed model. **D**. Cell-cell interactions between myeloid populations and non-myeloid cells as quantified using the cross- pair correlation function (cross-PCF). **E**. Cell-cell interactions made by CD8 and HIF1+ immune populations with other populations as quantified using the cross- pair correlation function. *p<0.05 versus complete spatial randomness (where cross-PCF score would equal 1). Box plots show range, interquartile range, and median of data (**B**, **C**).

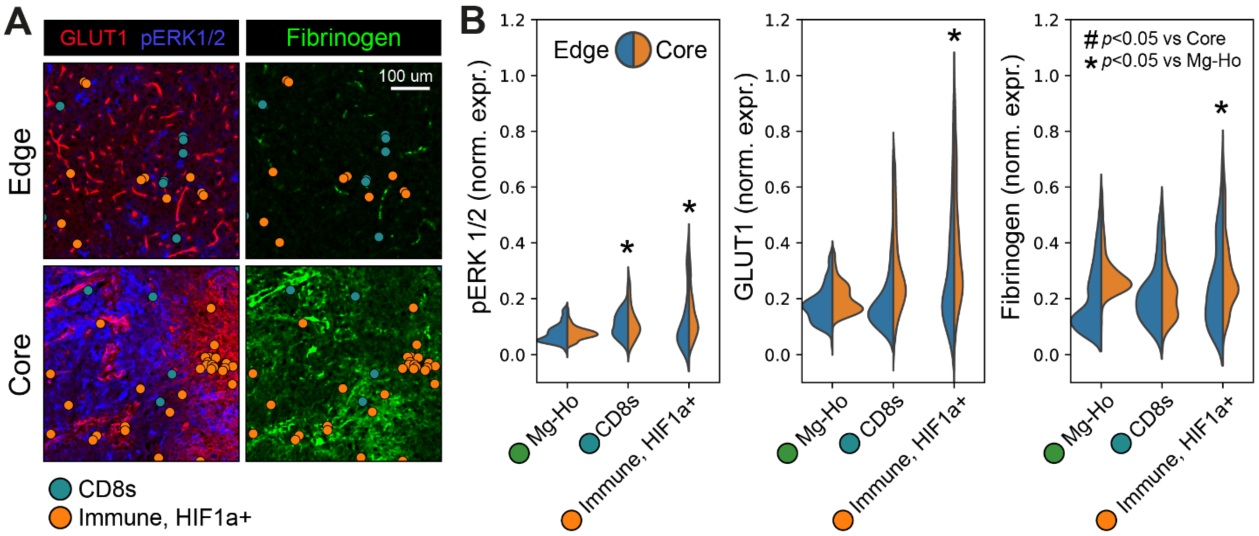

Supplemental fig. S4

A. CD8 and HIF1a+ immune populations mapped alongside markers of environmental hypoxia in GLUT1 and pERK1/2 staining, or fibrinogen. **B**. Quantification of environmental pERK1/2, GLUT1 and fibrinogen staining around each population in edge and core regions. A cell's environment was defined as a 40 µm square centred on the cell. Comparisons made by linear mixed models with Holm-Šídák corrections. Violin plot shows range and distribution of data.

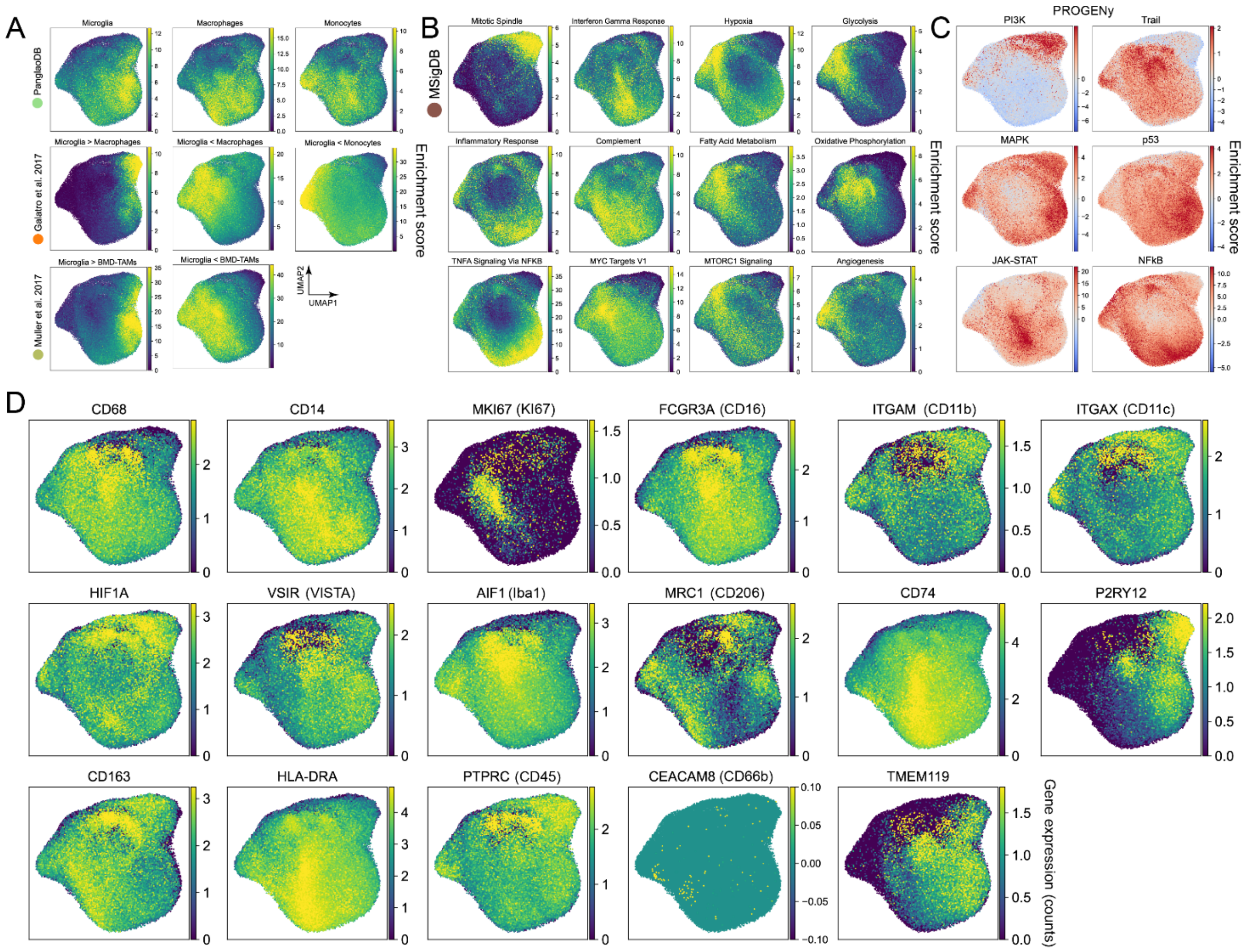

Supplemental fig. S5

Enrichment of specified gene lists was calculated by over representation analysis (ORA) for individual cells, which was then plotted by UMAP. **A**. ORA of gene lists differentiating microglia, macrophages, and monocytes (*6*, *55*, *80*). **B**. ORA of biological processes from the MSigDB database (*79*). **C**. Activation or suppression of signalling pathways assessed by reference to the PROGENy database (*70*). These analyses (**A, B, C**) were performed using the *Decouplr* package in Python. **D**. Expression of the genes encoding the myeloid proteins targeted by the IMC antibody panel.

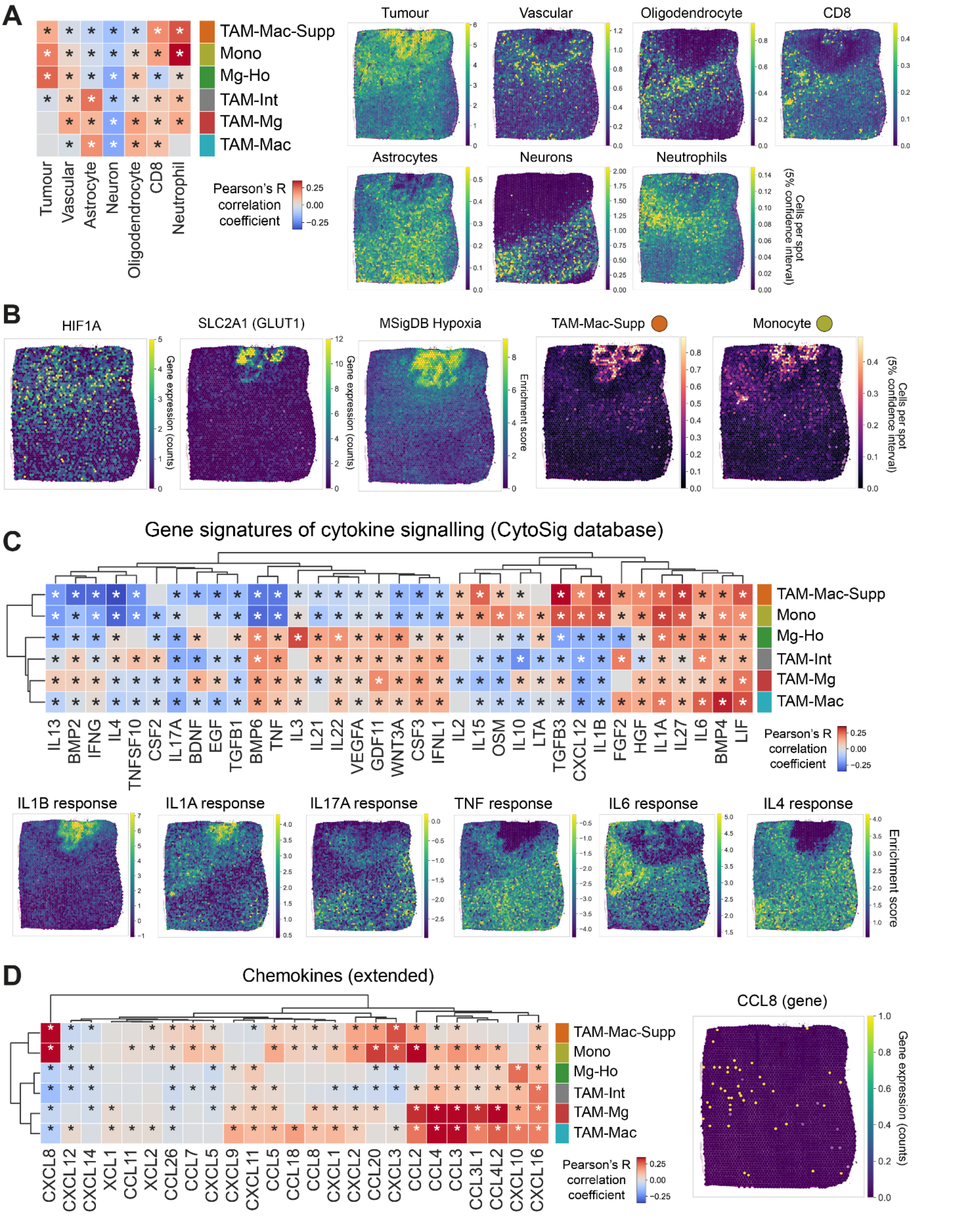

Supplemental fig. S6

A. Pearson's R correlation between the different myeloid populations and non-myeloid populations present in each spot. **B**. Representative ST case showing the distribution of the GLUT1 (SLC2A1) and HIF1A genes alongside the MSigDB hypoxia signature, and abundance of immunosuppressive macrophages (TAM-Mac-Supp) and monocytes. **C, D.** Myeloid cell abundances were correlated with the transcriptomic signatures of response to cytokine signalling from the CytoSig database (**C**) or individual chemokine genes (*Extended from* ***Figure 5J*** *to also include chemokines that that showed less variability between populations*). *p<0.05, with correction for multiple Pearson's R tests using the Benjamini-Hochberg procedure (**A, C, D**).

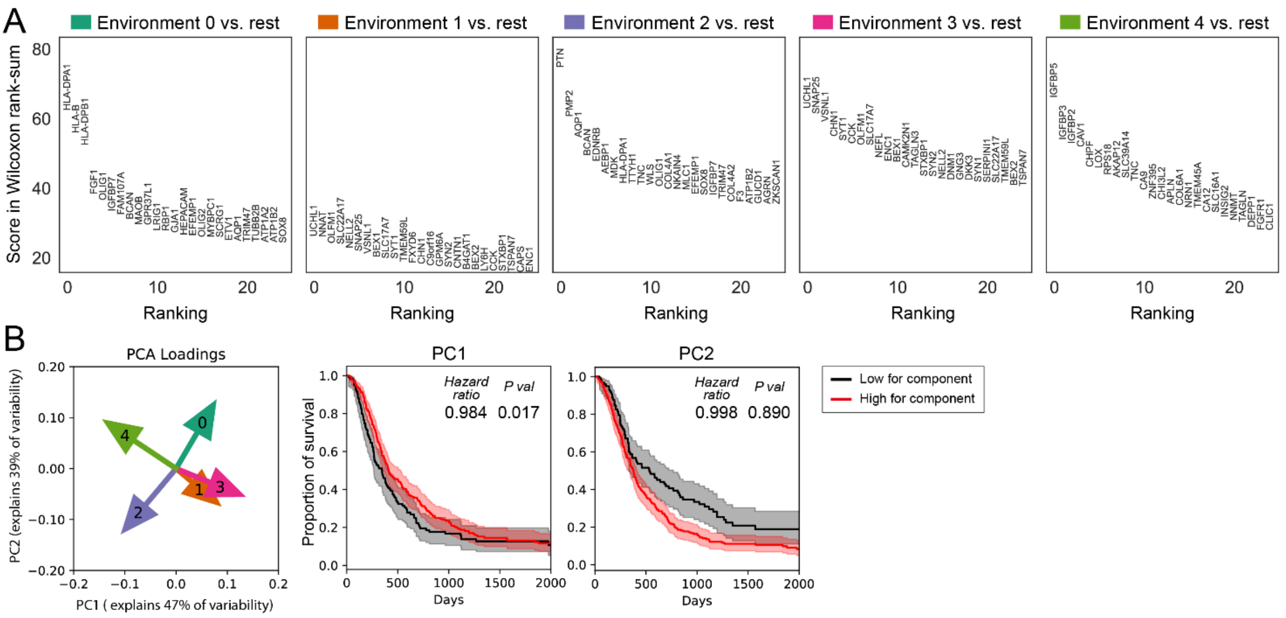

Supplemental fig. S7

A. The genetic changes associated with non-myeloid cells in each myeloid environment were extracted as described in **Figure 6E**. The top 25 differentially expressed non-myeloid genes differentiating each myeloid environment from the others was then calculated by Wilcoxon rank-sum analysis. **B**. Principal components analysis was used to summarise the abundance of the 5 myeloid environments into two principal components, which are by definition not correlated. The relationship between these principal components and GBM survival was modelled using Cox proportional-hazards, and top 50% and bottom 50% of patients for these PCs compared by Kaplan-Meier curves, shaded areas indicate 95% confidence intervals.
